# Combined effects of composting and antibiotic administration on cattle manure–borne antibiotic resistance genes

**DOI:** 10.1186/s40168-021-01006-z

**Published:** 2021-04-01

**Authors:** Ishi Keenum, Robert K. Williams, Partha Ray, Emily D. Garner, Katharine F. Knowlton, Amy Pruden

**Affiliations:** 1 Department of Civil and Environmental Engineering, Virginia Tech, 418 Durham Hall, 1145 Perry Street, Blacksburg, VA 24061 USA; 2 Department of Animal Sciences, School of Agriculture, Policy and Development, University of Reading, Reading, RG6 6EU UK; 3 Department of Civil and Environmental Engineering, West Virginia University, Morgantown, WV USA; 4 Department of Dairy Science, Virginia Tech, Blacksburg, VA USA

**Keywords:** Compost, Microbial community Succession, Thermophilic stress, Selection pressure, Antibiotic resistance

## Abstract

**Background:**

Research is needed to delineate the relative and combined effects of different antibiotic administration and manure management practices in either amplifying or attenuating the potential for antibiotic resistance to spread. Here, we carried out a comprehensive parallel examination of the effects of small-scale (> 55 °C × 3 days) static and turned composting of manures from dairy and beef cattle collected during standard antibiotic administration (cephapirin/pirlimycin or sulfamethazine/chlortetracycline/tylosin, respectively), versus from untreated cattle, on “resistomes” (total antibiotic resistance genes (ARGs) determined via shotgun metagenomic sequencing), bacterial microbiota, and indicator ARGs enumerated via quantitative polymerase chain reaction. To gain insight into the role of the thermophilic phase, compost was also externally heated to > 55 °C × 15 days.

**Results:**

Progression of composting with time and succession of the corresponding bacterial microbiota was the overarching driver of the resistome composition (ANOSIM; *R* = 0.424, *p* = 0.001, respectively) in all composts at the small-scale. Reduction in relative abundance (16S rRNA gene normalized) of total ARGs in finished compost (day 42) versus day 0 was noted across all conditions (ANOSIM; *R* = 0.728, *p* = 0.001), except when externally heated. *Sul*1, *int*I1, beta-lactam ARGs, and plasmid-associated genes increased in all finished composts as compared with the initial condition. External heating more effectively reduced certain clinically relevant ARGs (*bla*_OXA_, *bla*_CARB_), fecal coliforms, and resistome risk scores, which take into account putative pathogen annotations. When manure was collected during antibiotic administration, taxonomic composition of the compost was distinct according to nonmetric multidimensional analysis and *tet*(W) decayed faster in the dairy manure with antibiotic condition and slower in the beef manure with antibiotic condition.

**Conclusions:**

This comprehensive, integrated study revealed that composting had a dominant effect on corresponding resistome composition, while little difference was noted as a function of collecting manure during antibiotic administration. Reduction in total ARGs, *tet*(W), and resistome risk suggested that composting reduced some potential for antibiotic resistance to spread, but the increase and persistence of other indicators of antibiotic resistance were concerning. Results indicate that composting guidelines intended for pathogen reduction do not necessarily provide a comprehensive barrier to ARGs or their mobility prior to land application and additional mitigation measures should be considered.

**Video Abstract**.

**Supplementary Information:**

The online version contains supplementary material available at 10.1186/s40168-021-01006-z.

## Background

Antibiotic resistance is increasing in prevalence and economic costs, contributing substantially to human morbidity and mortality [1, 2]. Globally, antibiotic usage in livestock is expected to increase 67% by 2030 [3]. In 2018, 11.6 million kilograms of antibiotics were sold for food-producing animals in the USA [4]. Many antibiotics used in livestock are clinically relevant to human medicine. In particular, macrolides are characterized as “highest priority critically important antimicrobials” by the World Health Organization (WHO), while previous-generation cephalosporins, tetracyclines, lincosamides, and sulfonamides are classified as “highly important” [5], with all of these classes regularly used in the cattle industry. Until November 2017, beef cattle in the USA were administered antibiotic classes also used in humans for both therapeutic and growth promotion purposes [6]. However, the 2017 US Food and Drug Administration’s (FDA) Veterinary Feed Directive and WHO guidelines recommend restricting antibiotic use only for therapeutic purposes [7, 8].

Up to 100% of dosed antibiotics can be excreted as parent compounds, with the portion that is metabolized often remaining bioactive or converting back to the parent compound [9–11]. Excreted antibiotic residues can subsequently persist in manure, soil, and water [11, 12] and have measurable effects on resident microbial communities [13–15], including the potential to induce selection pressure even at low concentrations [16, 17]. Manure can also contain high loads of antibiotic-resistant bacteria (ARBs), even in the absence of antibiotic use, presumably because resistant gut commensal bacteria are passed from parent to offspring [18, 19]. In particular, amending soils with raw manure containing antibiotic residues has been observed to elevate levels of antibiotic resistance genes (ARGs), as well as mobile genetic elements (MGEs) involved in horizontal gene transfer, such as plasmids [20, 21]. Nonetheless, there are numerous benefits of applying manure-based amendments to soil, including improving soil texture, boosting nutrient levels, and reducing the need for chemical fertilizers, which incur a large carbon footprint during production [22]. Manure is also rich in microorganisms and resident ARBs can vary in their capacity to survive once applied to soil [23–25], which itself contains native ARB that can bloom in response to fertilizer [26]. Thus, it is critical to develop a comprehensive understanding of the effects of manure management practices, such as composting, on corresponding microbial communities and the total burden of ARGs that they carry (i.e., “resistome”) as a means of reducing/altering input of ARBs/ARGs to soil and passing on through the food chain.

Composting is often used to prepare manure for use as a soil amendment because it is beneficial for attenuating pathogen loads, reducing odors, and improving the quality of the soil [27]. Various composting guidelines are available, including those described in the FDA’s 2015 Food Safety Modernization Act (FSMA) [28], which requires a minimum of 3 days > 55 °C or 15 days > 55 °C for static or turned compost, respectively, but it is generally unknown whether there is a net benefit in terms of compost as a barrier to the spread of ARGs [29–32]. Hypothetically, composting could aid in attenuating antibiotic resistance by reducing fecal bacteria and overall microbial loads, while also degrading antibiotics [33–36], thus reducing selection pressures. A study conducted in parallel with the present study indicated that all administered antibiotics, except tylosin, were reduced dramatically (by 66 to 100%) during composting [36]. Conversely, the elevated microbial activity and thermophilic stress associated with composting could create conditions amenable to horizontal gene transfer or selection of resistant strains [37, 38], although some studies have reported limited evidence of ARG mobility during composting [39, 40]. A few studies have shown composting to be effective at reducing *Escherichia coli* carrying ampicillin, kanamycin, and tetracycline resistance, as well as *Acinetobacter* and Enterobacteriaceae carrying resistance to erythromycin and tetracycline in antibiotic-dosed swine manure, cattle manure and poultry waste [40–43]. Individual ARGs have been observed to increase or decrease during composting, depending on animal feed, animal type, use of fertilizers and other soil amendments, size of compost pile, and method of composting (static vs. turned) [14, 34, 44–46]. However, examining the resistome as a whole is needed to provide a more holistic understanding of effects of composting, including potential for horizontal gene transfer.

The objectives of this study were (1) to provide a comprehensive, parallel comparison of how composting affects the composition and mobility of ARGs in manure derived from cattle undergoing antibiotic administration, versus from control cattle, and (2) to determine the relative roles of microbial ecological succession versus the duration of the thermophilic phase in reducing the potential for antibiotic resistance to spread. Here we examined manures of dairy and beef cattle, which have relatively similar physiologies, but vary in antibiotics used and routes of administration. Dairy and beef manures were generated for composting following intramammary infusion of cephapirin and pirlimycin or in-feed sulfamethazine, chlortetracycline, and tylosin, respectively, versus from untreated cattle. Collection of manure during peak antibiotic excretion following standard administration practices and in a natural metabolized state, rather than exogenously spiking antibiotics, was a key aspect of achieving realistic conditions. Composting was performed in small-scale composters to enable multiple replicated and parallel comparisons of static versus turned composting methods. A trial of the static dairy compost conditions externally heated to 55 °C for 15 days further evaluated the effects of an extended thermophilic phase. Shotgun metagenomic sequencing was performed on a cross section of 60 samples to profile resistomes while quantitative polymerase chain reaction (qPCR) provided a sensitive measure of specific indicators of anthropogenic sources of antibiotic resistance across all 270 samples collected, including *sul*1, *tet*(W), and the class 1 integron integrase gene, *intI*1. 16S rRNA gene amplicon sequencing was also performed on every sample to characterize succession of the bacterial microbiota as they related to antibiotic administration, progression of composting, the extent of thermophilic phase, and the composition of corresponding resistomes. The findings here have key implications for both livestock and manure management strategies for limiting the potential for antibiotic resistance to spread.

## Methods

### Manure production

The methods for cattle selection, manure collection, and small-scale composting were described in a prior study focused on the fate of antibiotics [36]. In summary, manure for composting was procured from eighteen individually housed steers and cows selected for their respectively similar body weights, history of antibiotic use (none for steer, none in previous lactation cycle for cows), and consistent stage of lactation. All animal studies were approved under Virginia Tech IACUC protocols #13-145 and 14-262.

Nine Hereford steers were fed a basal diet of corn silage and medicated or non-medicated grain mix for seven days. Three steers were fed 350 mg of chlorotetracycline and sulfamethazine per day, three were fed 11 mg tylosin per kg feed, and three were fed a non-medicated diet. Feces and urine were collected from days 3 to 7 post-treatment. Nine dairy cows were selected for this study. Three peak lactation cows received no antibiotics, three peak lactation cows were treated with two intermammary doses of 50 mg pirlimycin; and three cows at the end of lactation received a single intermammary dose of 300 mg cephapirin per quarter (i.e. 300 × 4 = 1200 mg per cow), according to standard veterinary practice. Feces and urine were composited to obtain a homogenous mixture of “antibiotic” and “control” manure for both beef and dairy cattle (i.e., four distinct manures for subsequent composting) and to simulate the possibility of segregating antibiotic-containing manures as a management practice.

### Composting

#### Small-scale composting

The experimental setup and fate of antibiotics during composting were described previously [36]. In summary, the four different manures were mixed with alfalfa hay, pine bark mulch, and sawdust to adjust the C:N ratio to 24.5 and composted in triplicate using both static and turned composting methods, yielding 24 independent composters (wet mass = 20–22 kg). Static composters were aerated using an air pump and turned composters were turned daily. Samples were collected after 0, 4, 7, 14, 21, 28, 35, and 42 days of composting. On day 0, samples were also taken of each non-composted, raw manure. All samples were analyzed immediately for culturing and additional samples were stored at – 20 °C for molecular analysis.

#### Externally heated composting

An externally heated composting trial was employed to artificially impose a 15-day thermophilic phase using segregated aliquots of the same dairy manures used in the small-scale experiment. Composters were set up in 5-gallon barrels in triplicate for a total of 6 composters using the same compost feedstock recipe as the small-scale trial (stored at 4 °C prior to use). The compost was allowed to self-heat for the first 72 h, after which external heat tape was applied to maintain the thermophilic stage (>55°C) for 15 days. A mesophilic temperature range (35–45 °C) was then maintained for 3 weeks before allowing the compost to cool to room temperature. Samples were collected on days 0, 1, 3, 7, 14, 21, 28, 35, and 42 and raw manure samples were collected on day 0 for comparison with finished composts.

### Fecal coliform and *E. coli* enumeration

Ten grams of compost or manure were added to a sterile blender bag with 90 mL of 0.1% peptone solution to make a 1:10 dilution and mixed in a bag mixer for 2 min. A serial dilution was performed in 0.1% peptone solution prior to plating onto MacConkey agar and incubating for 24 h at 37 °C to enumerate total fecal coliforms (pink colonies; 30 and 300 CFU countable range). For *E. coli* quantification at higher specificity and sensitivity, the Colilert Quanti-Tray 2000 method (IDEXX, Westbrook, ME) was applied to 0.1% peptone solution serial dilutions weekly after day 21 to determine if the FSMA guideline for *E. coli* reduction had been achieved.

### DNA extraction

The FastDNA Spin Kit for Soil (MP Biomedicals, Solon, OH) served as the primary extraction kit applied to all samples. Following blending, 500 mg of compost or manure was aseptically transferred to an extraction tube. Extraction followed the manufacturer’s instructions, except that a 2-h incubation period was added to both protocols immediately following the bead-beating step to optimize lysis of microbial cells and the final centrifugation step was extended to 3 min to maximize capture of DNA. The OneStep PCR Inhibitor Removal Kit (Zymo, Irvine, CA) was applied to all DNA extracts.

### Metagenomic sequencing and analysis

Sixty representative DNA extracts were selected to provide a cross section among all manure types and the small-scale and externally heated compost conditions for metagenomic sequencing. DNA extracts were sequenced by the Biocomplexity Institute of Virginia Tech, Blacksburg, VA, on an Illumina HiSeq 2500 in High Output mode with 2 × 100 paired-end reads, with 12 samples pooled per lane across 5 lanes. Paired-end reads were annotated in MetaStorm using default parameters, with the amino acid identity (80%) aimed at preventing false positive annotations [47] and the *e*-value cutoff (1e−10) utilized to ensure lower quality matches were filtered out prior to assessment [48]. ARGs were annotated against the Comprehensive Antibiotic Resistance Database (CARD v1.0.6) [49] and plasmid-associated genes against the ACLAME database [50]. Given that differing annotation parameters and databases could produce different trends [51], resistomes of all samples were also annotated with DeepARG, which incorporates several publicly available databases and uses a deep learning algorithm to maximize ARG detection [47]. Relative abundances of total ARGs predicted by DeepARG were found to be strongly and significantly correlated with those annotated using CARD via MetaStorm, as described above (Fig. S1; Spearman’s *r* = 0.8, *p* <0.01). ARG richness was determined by enumerating each unique ARG detected by CARD and normalizing to the total million of reads for the sample.

Contigs were assembled in MetaStorm using IDBA-UD [52]. “Resistome risk,” defined as the cumulative potential for ARGs to occur on MGEs and in human pathogens, was calculated from the assembled contigs and compared among the samples using MetaCompare(v2 )[53]. Resistome risk is intended as a relative comparison among a similar sample set and is calculated from assembled metagenomic data as the product of the number of contigs containing an ARG, the number of contigs containing an ARG and MGE, and the number of contigs containing an ARG, MGE, and pathogen. Resistome risk is determined by annotating to an integrated ARG databases (CAR D[54], ARD B[55], MEGARe s[56], SAR G[57], and DeepARG-D B[47]), an integrated MGE databases (NCBI search for “integron” and I-VI P[58]), and a human pathogen database (WHO priority pathogens for ARG s[59]) with an *e*-value < 1e−10 and amino acid identity > 60%. To assess the potential influence of the assembly method on the observed resistome risk trends, all small-scale samples were also assembled using MEGAHIT [60] (Fig. S2). No significant differences were observed in resulting resistome risk scores (Wilcoxon, *p* = 0.5). On average, 48% of metagenomic reads per sample were successfully assembled into contigs using IDBA-UD. Contigs that occurred more than 3 times across all samples were further examined. Relative abundances were calculated by normalizing gene counts to abundance of 16S rRNA genes, annotated against the Greengenes database [61], factoring in target gene and 16S rRNA gene length as proposed by Li et al. [62].

### 16S rRNA gene amplicon sequencing

Two hundred and seventy DNA extracts from each composting trial were amplified via PCR targeting the V4 and V5 regions of the 16S rRNA gene following the online Earth Microbiome Project protocol using barcoded primers (515F/926R) [63]. Triplicate PCR products were composited, purified using a QIAquick PCR Purification Kit (Qiagen, Valencia, CA), and 240 ng of each composite was combined into 2 lanes of 150 samples each. Sequencing was performed on an Illumina MiSeq with V3 2 × 300 paired-end cycles. Reads were analyzed using the QIIME pipeline [64]. All singleton reads and chimeric sequences were removed and OTU tables were generated for taxonomic analysis. Samples were rarefied to 9052 base pairs. Jackknifed beta diversity analysis was performed to calculate unweighted and weighted UniFrac distance matrices for the comparison of taxonomic similarity.

### Quantitative polymerase chain reaction

qPCR was performed in triplicate on all DNA extracts using the CFX96 Touch Real-Time PCR Detection System (BioRad Laboratories, Hercules, CA) to quantify initial concentrations of 16S rRNA genes [65], *tet*(W) [66], *int*I1 [67], and *sul1* [68] using SsoFast Evagreen Supermix (BioRad Laboratories, Hercules, CA). A subset of DNA extracts were subjected to a dilution series and analysis by qPCR, based on which a dilution factor of 1:100 was selected and applied to all samples to minimize qPCR inhibition. All gene copy per gram measurements refer to compost at its actual moisture at the time of sampling. The ratio of wet to dry weight can be found in the SI published in Ray et al. [36].

### Statistical analysis

Statistical analyses were performed using R (3.4.1) [69]. Graphics were generated using Microsoft Excel and R packages ggplot2, cowplot, and RColorBrewer. Summary statistics were calculated using the ddply() function in the plyr package. A significance cutoff of *α* = 0.05 was applied. Statistical differences among gene abundances determined by qPCR were calculated using the Kruskal-Wallis nonparametric rank test and two-sided Wilcoxon rank sum tests. Nonmetric multidimensional scaling analysis (NMDS) and analysis of similarities (ANOSIM) were performed on unweighted UniFrac distance matrices for microbial communities and on Bray Curtis similarities using Primer 6 software [70] and the R package vegan. Spearman’s rank correlations were performed in JMP [71] to compare ARG profiles derived from metagenomic data and taxonomic class profiles from 16S rRNA gene amplicon sequencing data.

### Availability of data and materials

All unassembled metagenomic files and 16S rRNA amplicon sequences are available at NCBI BioProject PRJNA506850, https://dataview.ncbi.nlm.nih.gov/object/PRJNA506850, (corresponding SRAs by sample in Table S1: Metagenomics and Table S2: 16S rRNA Amplicons). Assembled metagenomes are available at https://bench.cs.vt.edu/MetaStorm/ under public project: “Antibiotic resistance on manure and compost methods.” MetaStorm sample identification can be found in Table S1.

## Results

### Small-scale compost

#### Temperature and coliform profiles

As described in Ray et al. [36], which reported the fate of antibiotics and other physicochemical parameters related to the composting process, static and turned compost conditions yielded similar temperature profiles, achieving 55 °C by day 2 and maintaining this thermophilic temperature until day 5. *E. coli* were reduced by the end of the study, at 42 days, but not below detection from either compost type (3-4 log MPN/g; Fig. S3).

#### Succession of microbiota during composting

**Fig. 1:**
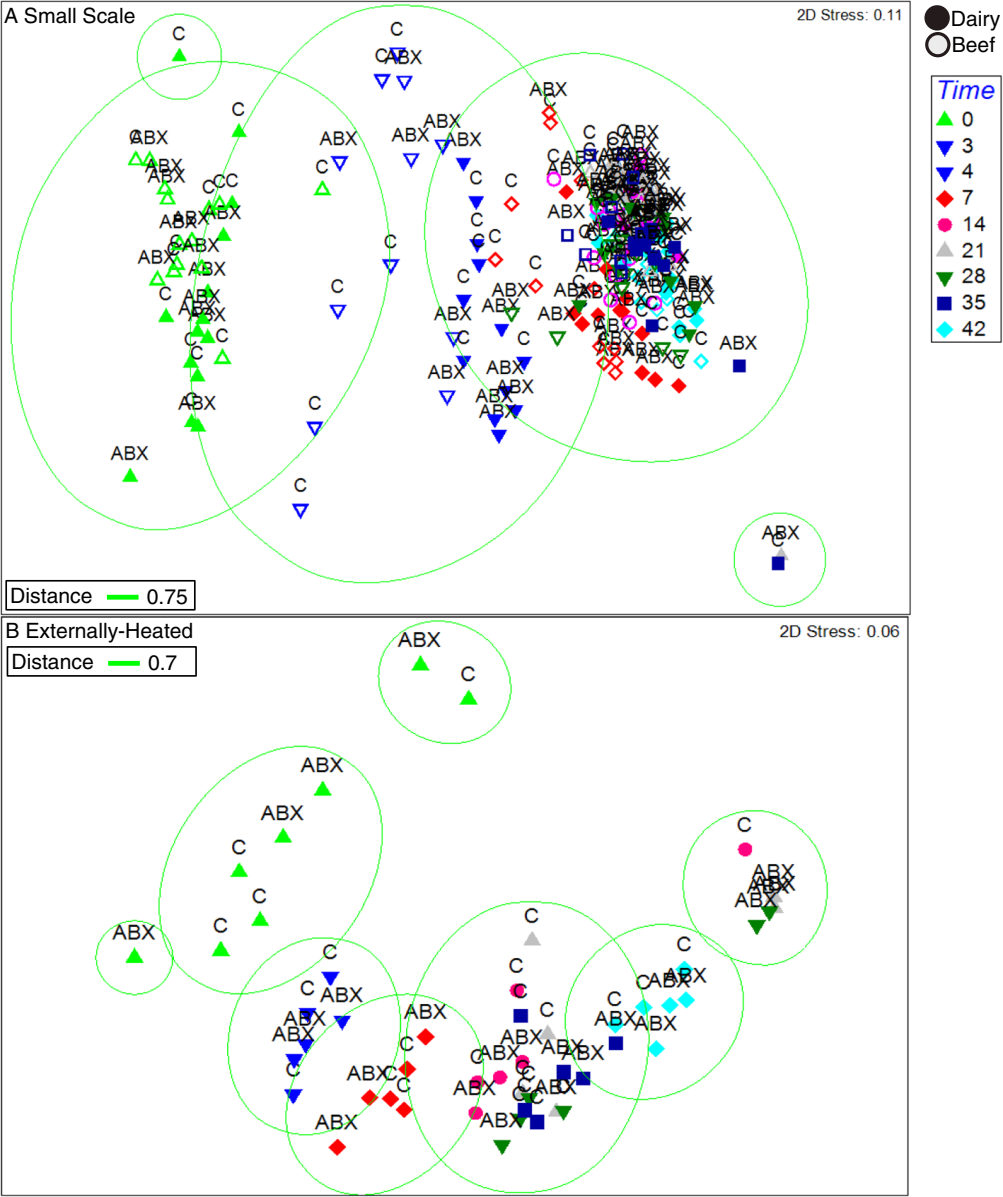
**a**, **b** Succession of bacterial microbiota, illustrated by nonmetric multidimensional scaling of unweighted Unifrac analysis of 16S rRNA gene amplicons, during composting of dairy (closed symbols) and beef (open symbols) manure, with antibiotic-administered (ABX) and control (C) cattle treatments indicated. Static and turned composting conditions were combined for this analysis. Similarity circles were drawn based on 75% similarity at the small-scale and 70% in the externally heated condition. Significant factors include **a** small-scale: duration of composting (ANOSIM; *R* = 0.5, *p* = 0.001), cattle (beef or dairy) (ANOSIM; *R* = 0.06, *p* = 0.002), compost type (static or turned) (*R* = 0.1, *p* = 0.001), and antibiotic administration (ANOSIM; day 0, beef: *R* = 0.3, *p* = 0.04; dairy: *R* = 0.4, *p* = 0.0008) and **b** externally heated: time (ANOSIM *R* = 0.7, *p* = 0.001)

Bacterial microbiota were profiled via 16S rRNA gene amplicon sequencing to obtain high sensitivity and taxonomic resolution across all 270 samples with time and capture succession patterns as composting proceeded through phases of intense organic matter biodegradation, thermophilic heating, and curing. Strikingly, taxonomic composition (NMDS analysis of unweighted UniFrac distances) clustered based on how many days the composting had progressed (Fig. 1, ANOSIM, *R* = 0.5, *p* = 0.002), rather than by cattle type, whether manure had been collected during antibiotic administration, or by the composting method employed. Presence/absence of antibiotic use for the feedstock raw manure significantly affected the composition of the initial microbiota in the beef and dairy conditions as well as the progression throughout composting across all samples and within the beef condition, though these did not play as large of a driving force (ANOSIM, day 0, beef: *R* = 0.3, *p* = 0.04; dairy: *R* = 0.4, *p* = 0.0008; all samples: *R* = 0.01864 , *p* = 0.012; beef: *R* = 0.03764 , *p* = 0.015). Throughout static and turned composting from day 0 to 4, in both the with and without antibiotic conditions, Bacilli significantly increased (Wilcoxon; *p* = 0.002), while the Clostridia (*p* = 0.002) and Methylacidiphilae (*p* ≤ 0.03) classes significantly decreased from their starting abundances (Fig. S4). Another apparent pattern was that the composition of the bacterial microbiota was more variable across the compost conditions on days 0 and 4 than on subsequent sampling days, where the composition converged to become remarkably similar across all conditions on the final sampling day (Fig. 1).

#### Succession of the resistome during composting

**Fig. 2:**
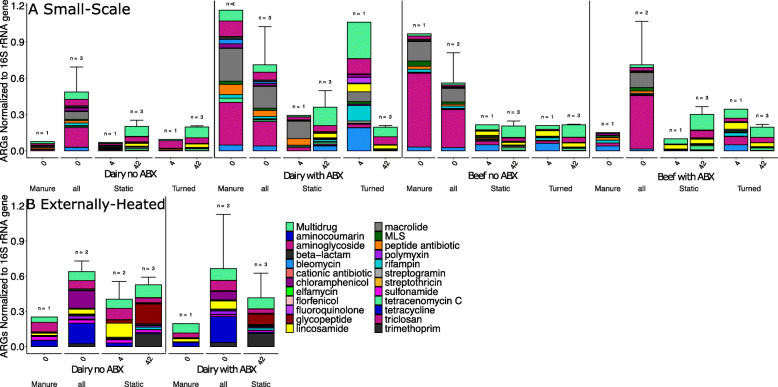
Average and standard deviation (error bars) of relative (normalized to total 16S rRNA gene reads) total ARG abundances by class for each compost experimental condition. ARG classes were identified by comparing Illumina shot-gun reads to CARD v1.0.4. **a** Small-scale. Across all samples/conditions day 0 > day 42 (*p* = 0.006). Further, day 0 > day 42 for all dairy conditions/samples combined (*p* = 0.004), all turned dairy conditions/samples combined (*p* = 0.04), and for all dairy with antibiotics (ABX) conditions/samples combined (*p* = 0.02). **b** Externally heated. No significant factors were found for total ARG relative abundance. In terms of composition of resistome determined by ANOSIM, duration of composting was a significant factor for both the small-scale (*R* = 0.161, *p* = 0.007) and externally heated (*R* = 0.2, *p* = 0.05) conditions. Externally heated dairy with ABX day 4 condition was not sequenced

Shotgun metagenomic sequencing was performed on 60 compost samples to characterize the resistome of the initial compost mixture (day 0), thermophilic phase (day 4), and the finished compost (day 42) (Fig. 2). Notably, a significant decrease in relative abundance (i.e., ARG copies per 16S rRNA gene copies) of total ARGs across all compost conditions was observed from day 0 to day 42 (Fig. 2, Wilcoxon, *p* < 0.001), indicating that the composting process generally imposed negative selection pressure on bacteria carrying ARGs. There was no significant difference in total ARG relative abundance among the four raw manure types (Kruskal-Wallis, *p* = 0.3), among the different initial day 0 compost mixtures (Kruskal-Wallis, *p* = 0.7), or among the different finished compost types (Kruskal-Wallis, *p* = 0.7).

The range of ARG alpha diversity in terms of unique ARGs identified (i.e., richness) in all samples was 1.76–12.8 ARGs/million reads, with an average of 6.4 ARGs/million reads. Significant decreases in diversity were observed in the dairy with antibiotic condition for both composting methods with time (Kruskal-Wallis; *p* = 0.004; Fig. S5). Otherwise, ARG diversity did not measurably change from day 0 to day 42 for any of the other conditions.

**Fig. 3:**
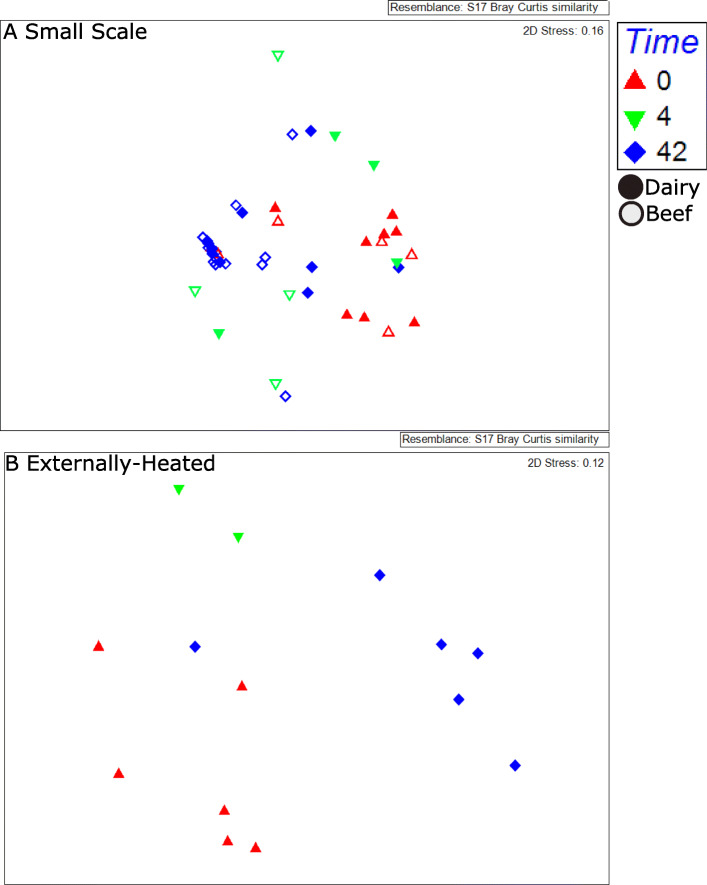
**a**, **b** Succession of resistomes with time during composting of dairy (closed shapes) and beef (open shapes) manure as illustrated by nonmetric multidimensional scaling of Bray Curtis distances of ARG annotations. Antibiotic versus no-antibiotic conditions are not labeled because there were no significant differences for either manure type or time point. Significant factors included **a** small-scale: duration of composting (*R* = 0.4, *p* = 0.001) and compost type (static or turned) (*R* = 0.1, *p* = 0.008) and **b** externally heated: duration of composting (*R* = 0.7, *p* = 0.001)

NMDS analysis of ARG profiles (i.e., types and relative abundances of ARGs) provided a broad comparison of the resistomes among the compost conditions (Fig. 3). Contrary to what was observed for the composition of the microbiota, no effect was observed on the finished compost as a function of antibiotic dosing (ANOSIM; *R* = − 0.05 *p* = 0.8). There was also no significant difference in initial ARG profiles among the four uncomposted manures used in this study (ANOSIM; *R* = 0.05, *p* = 0.4) or between these manures and their corresponding day 0 compost mixtures (ANOSIM; *R* = 0.07, *p* = 0.4). Composting, on the other hand, significantly shaped ARG profiles (Fig. 3). While there was no initial difference in day 0 compost, by day 42, there was a significant effect of composting method (static versus turned) (*R* = 0.4, *p* = 0.0004), while manure type did not have an effect (ANOSIM; *R* = − 0.05, *p* = 0.8). The shift in ARG profile in the compost as a function of time was significant (ANOSIM; *R* = 0.4, *p* = 0.0001), but the effect was not as strong as that observed for the succession of the microbiota.

#### Assessing resistome risks associated with composting

**Fig. 4:**
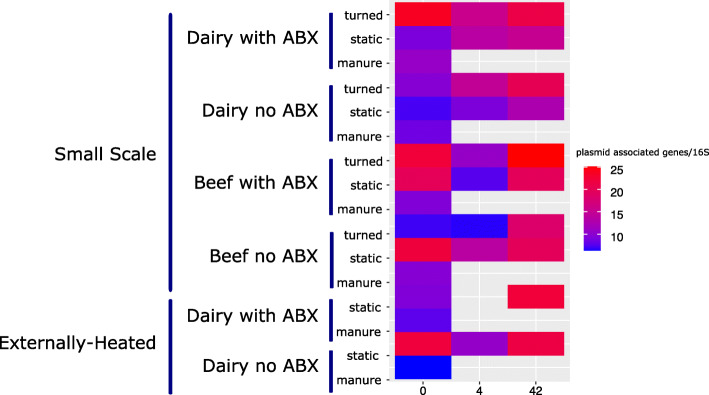
Relative abundance of plasmid-associated genes annotated using the ACLAME database. Within the beef and dairy small-scale experiments, numbers increased significantly with time (*p* = 0.03, *p* = 0.02). Values plotted are averages, with *n* varying from 1 to 3 (see Fig. 2)

Plasmid-associated genes were also analyzed as a key indicator of the mobility of the resistome and, in general, were found to increase in relative abundance with time regardless of cattle type or antibiotic dosing condition (Fig. 4, Wilcoxon, beef: *p* = 0.03, dairy: *p* = 0.02).

**Fig. 5:**
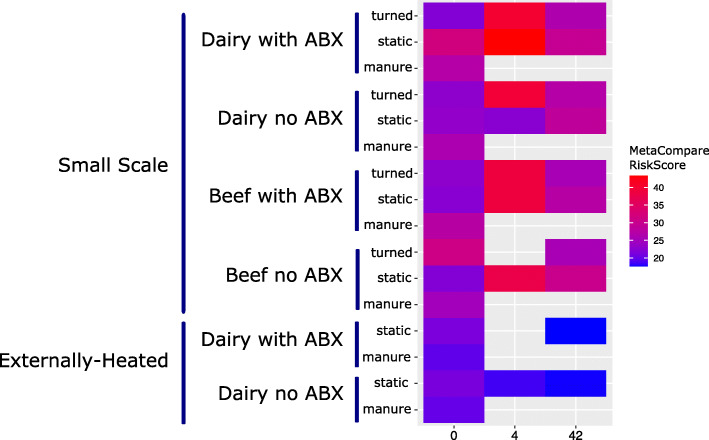
Heatmap of MetaCompare resistome risk scores determined from all available metagenomics data sets. Factors that changed significantly with duration of composting: small-scale: all conditions/samples combined (*p* = 0.0007), dairy conditions/samples combined (*p* = 0.02), dairy turned condition (*p* = 0.03). Externally heated: Both dairy with and without antibiotics decreased (*p* = 0.04, *p* = 0.04). Values plotted are averages of *n* varying from 1 to 3 (see Fig. 2)

To gain insight into the extent that ARGs occurred on MGEs and/or in pathogens, a resistome risk assessment was carried out using MetaCompare [53]. Here, resistome risk [72] is defined as a relative ranking system in which contigs assigned to taxa known to contain pathogens and annotated with ARGs and MGEs are considered to represent the greatest relative risk. The general pattern across all conditions was an increase in the risk score during the thermophilic phase, followed by a decrease to a level similar to the initial condition by day 42 (Fig. 5). Changes in resistome risk score with time were statistically significant for all dairy conditions combined and the dairy turned condition (Fig. 5, Kruskal-Wallis; all dairy samples: *p* = 0.02, dairy turned condition: *p* = 0.03). Notably, there were 39 pathogen/ARG/MGE contigs that persisted through composting. These were predicted to originate from *Staphylococcus*, *Streptococcus*, and *Vibrio*, encoding resistance to a wide array of antibiotics (e.g., aminoglycoside, sulfonamide, and trimethoprim) and being carried on a diverse range of transposases, recombinases, and plasmids (Fig. S6). No differences were observed in the distribution of the 39 contigs in the finished compost as a function of whether or not the cattle had been administered antibiotics.

In terms of individual classes and mechanisms of resistance, the abundance of ARGs corresponding to trimethoprim, tetracycline, and macrolide-lincosamide-streptogramin (MLS) resistance classes decreased during composting to a striking extent (Fig. 2). Only two resistance classes, bleomycin and beta-lactam, were found to positively correlate with any taxonomic group (Spearman, *p* < 0.05), with the Firmicutes phyla and the Erysipelotrichia class, respectively. These are both gut commensal-associated microbiota and thus expected to decrease during composting due to the elevated temperatures and microbial community succession.

**Fig. 6:**
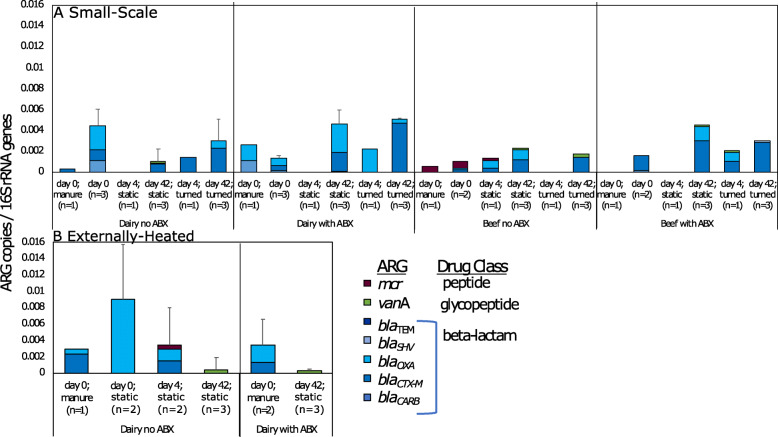
Clinically relevant ARGs for all conditions (clinically relevant ARGs selected for this analysis are listed in Table S3). Significant increases with time at the small-scale included all conditions/samples combined (*p* = 0.002, Kruskal-Wallis) and all beef conditions/samples (*p* = 0.02). Significant decreases with time for externally heated included all dairy conditions/samples (*p* = 0.006) and within the dairy with antibiotic condition (*p* = 0.04)

A “clinically relevant” subset of ARGs, defined as genes that convey resistance to antibiotics currently used in clinical settings, was compiled and subject to separate comparison (Fig. 6, Table S3). Notably, clinically relevant ARGs were found to collectively increase with time as composting progressed (Kruskal-Wallis, *p* = 0.002), with beta-lactam ARGs being particularly dominant. Interestingly, although this class of antibiotics was only administered to the dairy cows, there was no difference in detection of the corresponding ARGs in terms of a time-paired comparison between beef and dairy experiments (Kruskal-Wallis, *p* = 0.5).

#### Response of antibiotic resistance indicator genes during composting

**Fig. 7:**
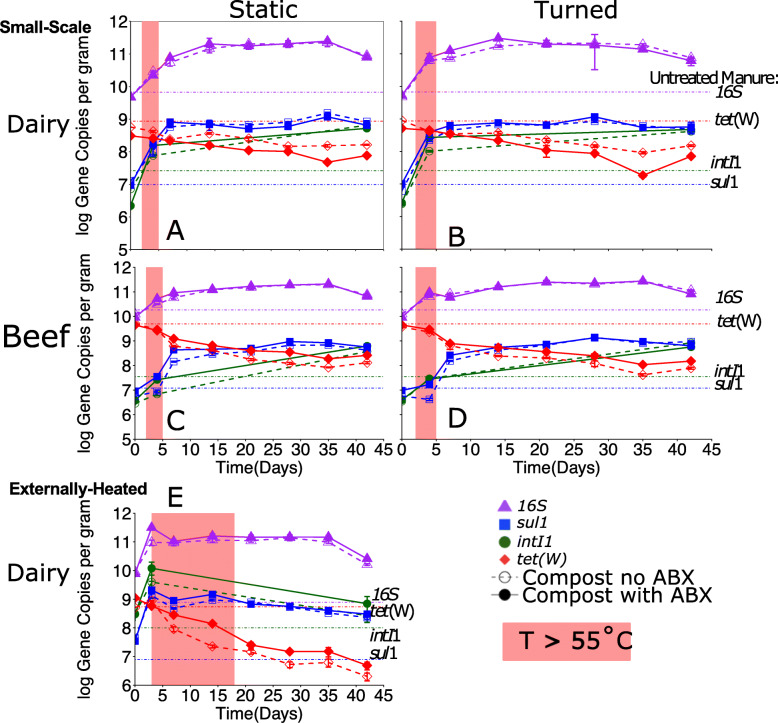
**a–f** Absolute abundance of *sul*1, *tet*(W), 16S rRNA gene copies and *intI*1 genes for all scales by cattle type, antibiotic (ABX) dosing, and composting method. The shaded portion of the plot indicates the duration of the thermophilic phase (> 55 °C). Error bars represent standard deviation of three biological replicates. All significant differences can be found in SI Table 6. Noteworthy differences include **a–d** small-scale: decrease in *tet*(W) from day 0 to day 42 (*p* = 0.001–0.02), increase in *sul*1 from day 0 to day 42 (*p* = 0.0004–0.02), and increase in *int*I1 from day 0 to day 42 (*p* = 0.0004–0.008,) and **e** externally heated: decrease from day 0 to day 42 in *tet(W* (*p* = 0.008) and *sul1*increase(*p* = 0.004)

Notably, *sul*1, as measured by qPCR, absolute abundance (i.e., gene copies per gram) increased during composting across all experimental conditions (Fig. 7, Fig. S7, Kruskal-Wallis, *p* = 0.004-0.03). Similarly, *int*I1 absolute abundance also increased across all conditions (Kruskal-Wallis, *p* = 0.004). Further, *sul*1 and *int*I1 correlated strongly and positively (Spearman’s *ρ* > 0.72, *p* < 0.05), as would be expected based on the common insertion of *sul*1 in the class 1 integron [73].

Absolute and relative abundances of *tet*(W) decreased across all compost conditions from day 0 to day 42 (Fig. 7, Fig. S7, Kruskal-Wallis, *p* = 0.003). Notably, *tet*(W) was the only of the three indicator ARGs for which the reduction pattern was influenced by whether the manure was collected from antibiotic-treated cattle. Counterintuitively, *tet*(W) decreased faster in the dairy with antibiotic than the dairy without antibiotic condition (Fig. 7). However, when beef manure was composted, *tet*(W) decreased faster in the control than in the antibiotic manure.

### Externally heated compost

#### Antibiotic, temperature*, and coliform profile*

The externally heated compost trial was carried out to extend the thermophilic stage and gain insight into the effect of elevated temperature versus natural progression of the compost on the compost microbiota and resistome. As expected, *E. coli* were successfully reduced below detection by the extended thermophilic period (Table S4). Further, pirlimycin decayed in a pattern similar to that observed at the small-scale [36], with more than 99% removed by day 7 (Table S5).

#### Succession of the microbiota with time

As was observed at the small-scale, NMDS analysis revealed duration of composting to be the overarching driver of the composition of the bacterial microbiota in the externally heated composts (ANOSIM; *R* = 0.5, *p* = 0.0001) (Fig. 1). The dominant taxonomic classes in the externally heated condition shifted to a lesser degree than at the small-scale (Fig. S4). Similarly, Bacilli increased as Clostridia decreased and Actinobacteria increased on day 3. The bacterial taxonomic profile of externally heated versus small-scale composts was significantly different when comparing across all time points (ANOSIM; *R* = 0.5, *p* = 0.001), as well as on the final day of composting (ANOSIM; *R* = 0.999, *p* = 0.001). The final compost from the externally heated condition was also more taxonomically diverse than the final compost at the small-scale (*p* = 0.02, Wilcoxon).

#### Succession of the resistome

Contrary to the small-scale trial, there was no significant reduction in relative abundance of total ARGs as a result of externally imposing a 15-day thermophilic period (Fig. 2). The relative abundance of total ARGs in the externally heated compost tended to be greater compared with small-scale compost (Wilcoxon; *p* = 0.055), although NMDS analysis did not reveal a difference in resistome composition between the finished small-scale and externally heated composts. Consistent with the small-scale composting, ARG profiles did not vary as a function of antibiotic treatment (ANOSIM; day 0: *R* = 0.5, *p* = 0.1; day 42: *R* = 0.6, *p* = 0.1), but did shift with time (Fig. 3, ANOSIM; *R* = 0.7, *p* = 0.001). The richness of ARGs did not vary with time, nor did plasmid-associated genes or clinically relevant ARGs (Figs. 3, 4 and 6). Notably, relative resistome risk scores were markedly reduced in externally heated composts compared with the small-scale composts (Fig. 5). Only three contigs were identified as containing pathogen-like/ARG/MGE DNA (Fig. S7). These again corresponded to *Vibrio* and *Staphylococcus* conveying aminoglycosides and bleomycin ARGs, respectively.

Consistent with the small-scale composting, absolute abundances of *sul*1, *intI*1, and 16S rRNA genes generally increased and *tet*(W) decreased during externally heated composting (Fig. 7). This was the case in all but the dairy condition with no antibiotics for *sulI*1 (*p* = 0.01) and for *tet*(W) for all conditions from time 0 to day 42 (*p* = 0.01)*.* Relative *sul*1, *tet*(W), and *intI*1 abundance (Fig. S7) all were significantly different on day 42 compared with day 0 (*p* = 0.01), with *int*I1 and *sul*1 increasing and *tet*(W) decreasing.

## Discussion

Given that the majority of antibiotics sold in the USA and many parts of the world are administered to livestock, it is important to understand how this affects the carriage of ARGs in corresponding manures and if agricultural management practices, such as composting, act to amplify or attenuate their potential to spread downstream to environmental, food, and human receptors. Here, we carried out a comprehensive integrated study to assess effects of different composting approaches on manure collected during standard antibiotic administration, versus without antibiotic treatment, for both beef and dairy cattle. We utilized a variety of measurements to gain a mechanistic understanding into how the composting process affects the resistome and to evaluate the potential for antibiotic resistance in the finished compost to spread and pose a human health risk through association with pathogens.

In general, composting had a strong effect in shaping the microbiomes of finished composts and both resistome and microbiota profiles tended to converge across conditions, regardless of whether the manure was collected from beef or dairy cattle or if antibiotics had been administered.

The overall results indicate that the natural microbial ecological succession that occurs during composting likely plays a major role in dictating the composition of the resulting resistomes. The specific microbial taxa that shifted during composting was logical, with Clostridia being strict anaerobes and highly characteristic of cattle manure, thus their reduction with (aerobic) composting is as expected [74–76], while Bacilli are found both in cattle manure and soil and are highly diverse and facultative and thus capable of increasing during composting [77, 78]. Although progression of composting was also the strongest factor for the externally heated trial, a distinct trajectory was observed in the composition of the microbiota and resistome relative to small-scale. A key result was that the relative abundance of total ARGs decreased during composting at the small-scale, but not in the externally heated composting trials. This is an important finding, indicating that it is the natural composting process, rather than the thermophilic temperature itself, that acts to reduce total ARG relative abundance across the bacterial community.

*Tet*(W) was reduced universally in all compost treatments, including both small-scale and externally heated trials. The decrease in *tet*(W) in response to composting is consistent with the observations of Selvam et al. [79] and Storteboom et al. [44], suggesting that this ARG is generally sensitive to composting. However, *sul*1 and *intI*1 both increased in relative and absolute abundance across all composting conditions. *sul*1 and *intI*1 are becoming widely applied as sensitive indicators of anthropogenic sources of antibiotic resistance and potential to spread [73]. In particular, their association with class 1 integrons, which have been found among taxonomically diverse bacteria and can carry and mobilize multiple ARGs [58, 80], presents a concern that composting may not fully eliminate the potential for antibiotic resistance to spread.

Metagenomic analysis of resistomes further revealed increases in several clinically relevant ARGs during composting, especially those encoding resistance to beta-lactams. While pirlimycin and tylosin, two of the antibiotics administered to the cattle, belong to the MLS drug class, ARGs encoding resistance to this class markedly decreased throughout composting, suggesting that there was little selection pressure. However, there were indications that the resistome may become more mobile during composting, enabling the spread of ARGs to new host organisms. In particular, plasmid-associated genes increased measurably in all composting conditions, including the externally heated trial. Resistome risk scores, which provided a relative comparison across samples of associations of ARGs with MGEs and taxonomic groups containing pathogens, increased initially during the thermophilic phase of small-scale composting, but then decreased back to levels similar to the initial condition by day 42. This suggests that the shock of the thermophilic phase might induce horizontal gene transfer of ARGs. However, with the extended thermophilic phase of 14 days, resistome risk scores attained the lowest levels observed throughout the study, as was indicated by substantially fewer contigs annotated as containing ARGs, MGEs, and pathogen-like sequences (Fig. S4). Clinically relevant ARGs were also more effectively reduced by the extended thermophilic phase, as were fecal coliforms and *E. coli*. These results are consistent with the known benefits of an extended thermophilic phase for pathogen reduction, but this study suggests that the benefits of time × temperature guidelines intended for diminishing fecal pathogen risk do not translate to reducing the total ARG load or their potential to mobilize.

Whether the manure was collected during antibiotic administration had surprisingly little effect on indicators of antibiotic resistance. Notably, there was no significant difference in initial resistome composition among the four manures (i.e., dairy with antibiotics, dairy control, beef with antibiotics, beef control) prior to composting (ANOSIM, *p* > 0.05). Similarly, no differences were found in the finished composts in terms of overall resistome composition, relative abundance of total ARGs, clinically relevant ARGs, plasmid-associated ARGs, or resistome risk scores as a function of whether the manure had been collected during antibiotic administration. The only discernable differences based on antibiotic administration were with respect to *tet*(W), ARG alpha diversity, and the composition of the bacterial microbiota. Specifically, *tet*(W) decayed faster in the dairy antibiotic compost and slower in the beef antibiotic compost than in their respective controls. This could relate to the fact that chlortetracycline was administered to the beef cattle and thus continued to exert some selection pressure for bacteria carrying *tet*(W). In the case of the dairy manure, *tet*(W) reduction may have been enhanced by pirlimycin killing bacteria carrying *tet*(W), assuming that *tet*(W) did not co-occur with ARGs conferring resistance to pirlimycin. ARG alpha diversity was the only other antibiotic resistance indicator that varied by antibiotic administration, significantly decreasing during composting only in the dairy with antibiotic condition. According to NMDS analysis of bacterial 16S rRNA gene amplicons, taxonomic composition of both beef and dairy composts was influenced by whether antibiotics were administered across all timepoints. This brings to light an interesting point that the taxonomic composition of the compost was actually more sensitive to antibiotic administration than the composition of the resistome. Overall, the results indicated that antibiotic administration was a minor factor shaping the composition of the resistome compared with composting itself.

Finally, it is acknowledged that metagenomic analysis is an evolving field, particularly for the characterization of antibiotic resistance in complex environmental samples [51, 81, 82]. Here we demonstrated that overall trends in total ARG relative abundance held true when using a distinct deep learning–enabled pipeline (DeepARG) for ARG annotation (Fig. S1) or a different assembler (MEGAHIT) for resistome risk comparison (Fig. S2). Still, it is to be expected that precise conclusions will vary to some degree as a function of the parameters of the analysis, depending on the focus of the study (e.g., behavior of specific classes/types of ARGs and identification of neighboring genes). Future efforts towards continuing to standardize and validate metagenomic analysis approaches are warranted.

## Conclusions

The comprehensive comparative nature of this study provides new insight into the relative benefits of various on-farm management approaches in terms of their potential to control the spread of antibiotic resistance. The study was designed in such a manner to inform with respect to whether typical antibiotic use in cattle poses special concerns for the control of antibiotic resistance in resulting manure, and if such manures merit segregation and specialized treatment relative to antibiotic-free manures. Surprisingly, there were few discernable effects as a function of antibiotic administration, suggesting that the cattle used in this study already carried a robust gut resistome that was not substantially affected by antibiotic use. Lack of difference in antibiotic-treated and control livestock in manure-borne ARGs has been noted in other studies [83, 84]. Composting, on the other hand, had a strong and overarching influence on the cattle manure resistomes. However, while some metrics of antibiotic resistance substantially reduced, such as the relative abundance of total ARGs and *tet*(W), others persisted or even increased. *sul1* and *intI*1 are widely being considered monitoring targets for assessing anthropogenic sources of antibiotic resistance and efficacy of mitigation measures [85]. Notably, in this study, the increase of these two genes during composting was consistent with increases in plasmid-associated genes and beta-lactam ARGs, suggesting that they may be good monitoring targets when comprehensive measurements such as those employed in this study are not possible. With respect to the benefits of an extended thermophilic phase during composting, this study yielded mixed results. Pathogen indicators and resistome risk were reduced, but total ARGs, *sul*1, *int*I1, and indicators of mobility were not. Further, little to no difference was observed between turned and static composting conditions, suggesting that, from an antibiotic resistance standpoint, there may be little value in the extra effort required to turn and maintain compost heated for extended periods. Composting is well known and effective at reducing microbial pathogens and produces a consistent product, but additional mitigation measures are advisable to minimize the potential for antibiotic resistance to spread along the farm-to-fork continuum. Additional barriers beyond composting, such as imposed distances from water bodies for land application and wait periods before harvest would be wise to consider.

## Supplementary Information

**Additional file 1.**

## Data Availability

All unassembled metagenomic files and 16S rRNA amplicon sequences are available at NCBI BioProject PRJNA506850, https://dataview.ncbi.nlm.nih.gov/object/PRJNA506850, (Corresponding SRAs by sample in Table S1: Metagenomics and Table S2: 16S rRNA Amplicons). Assembled metagenomes are available at https://bench.cs.vt.edu/MetaStorm/ under public project: “Antibiotic resistance on manure and compost methods.” MetaStorm sample identification can be found in Table S1. The culture and qPCR datasets can be made further available upon request to the corresponding author.
