# Supplementary material for: Combined effects of composting and antibiotic administration on cattle manure–borne antibiotic resistance genes
Source: Microbiome. 2021 Apr 1;9:81. doi: 10.1186/s40168-021-01006-z (PMC8017830; doi:10.1186/s40168-021-01006-z)
Supplement: Supplementary file 2 — Additional file 1. [file 40168_2021_1006_MOESM2_ESM.pdf]

Title: Combined Effects of Composting and Antibiotic Administration on Cattle Manure-borne Antibiotic Resistance Genes

Ishi Keenum*[1], Robert Williams*[1], Partha Ray[2], Emily Garner[1,3], Katharine Knowlton[4], Amy Pruden[1]

Supplemental Information

*These authors contributed equally to this work

[1] Department of Civil and Environmental Engineering, Virginia Tech Blacksburg, VA

[2] Department of Animal Sciences, School of Agriculture, Policy and Development, Univ. of Reading, Reading RG6 6AR, UK

[3] Department of Civil and Environmental Engineering, West Virginia University, Morgantown, WV

[4]Department of Dairy Science, Virginia Tech, Blacksburg, VA, USA

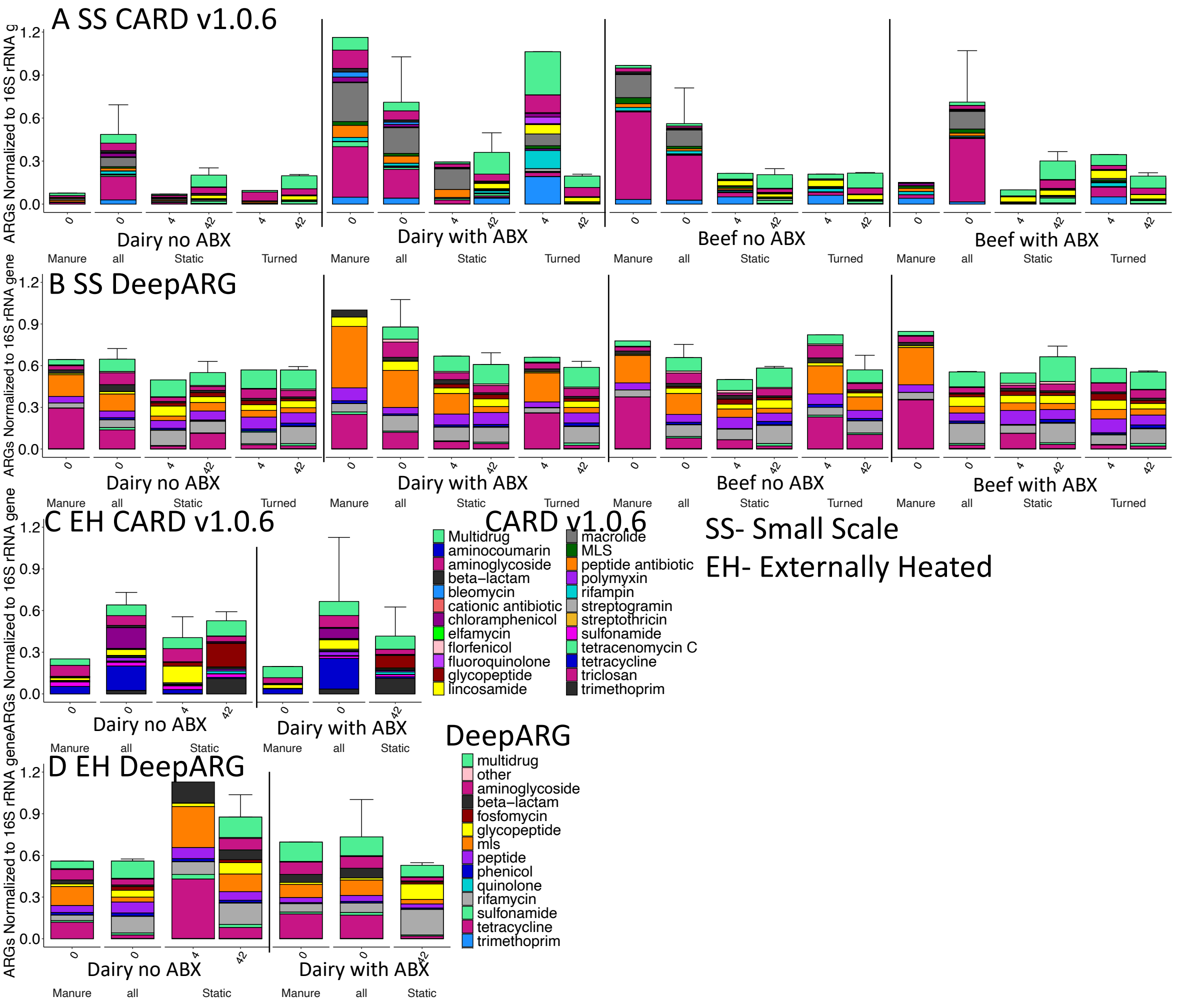

Fig. S1 Comparison of ARG annotation with CARD v1.0.6 via MetaStorm and DeepARG using the DeepARG short reads pipeline. Annotation to CARD was applied conservatively, to ensure confidence in ARGs detected, while DeepARG employs deep learning to maximize detection of potential ARGs. A) Small-Scale condition annotated via MetaStorm to the CARD v1.0.6 database normalized to 16S rRNA genes. B) Small-scale (SS) condition annotated with ARGs predicted via the DeepARG pipeline normalized to 16S rRNA genes. C) Externally-heated (EH) condition annotated via MetaStorm to the CARD v1.0.6 database normalized to 16S rRNA genes. D) Externally-heated condition with ARGs predicted via DeepARG pipeline normalized to the 16S rRNA genes. Time 0 composts were combined for static and turned conditions at the small scale as they were generated at the same time from the same starting materials. For both methods, amino acid identity ≥80% and e-value cutoff = 1e-10. Additionally, in DeepARG, the probability for considering reads to be ARG-like = 80%. Given that the categories of ARGs annotated differ by the two methods (fewer ARG categories in DeepARG), relative abundance of total ARGs annotated by both methods were compared. **Relative abundances of total ARGs were found to be strongly and significantly correlated (Spearman, r= 0.8, p < 0.01)**. Error bars are shown in cases where n≥3.

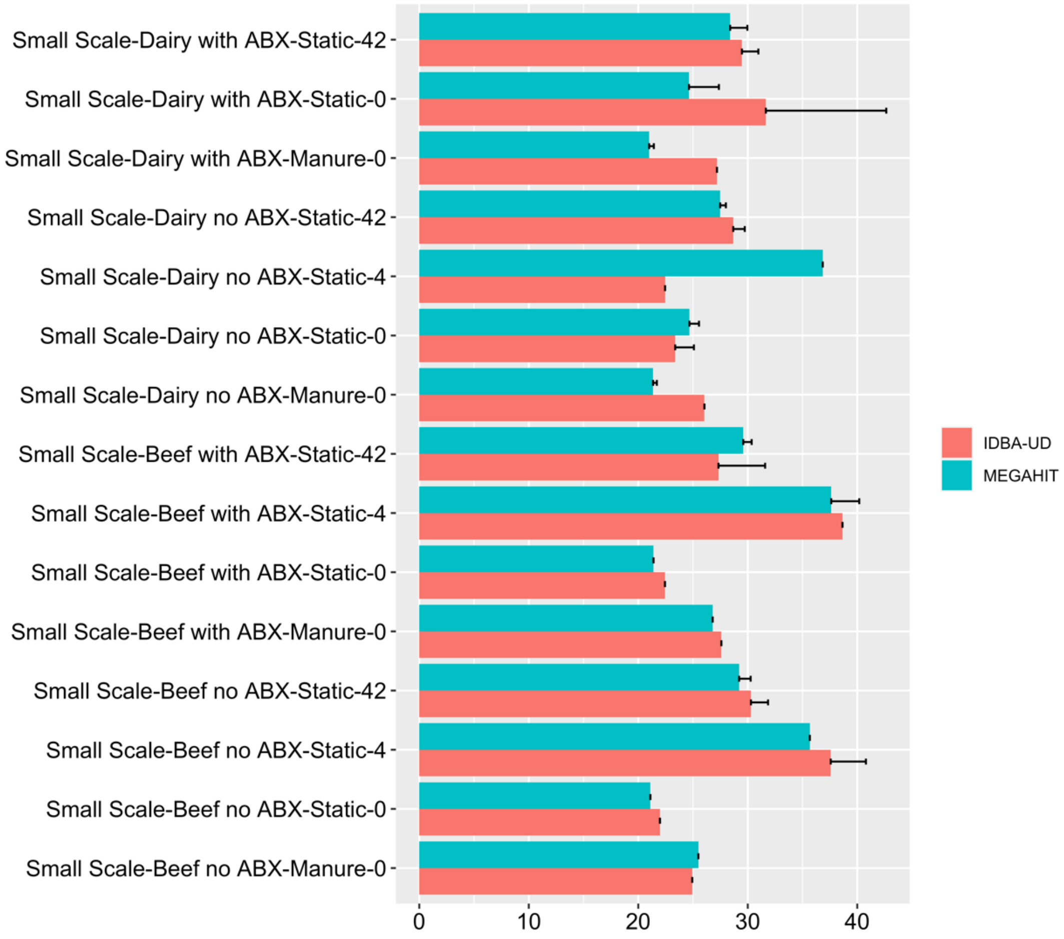

Fig. S2 Comparison of MetaCompare Risk Scores via two Assemblers. No significant differences were observed in resistome risk scores when determined using IDBA-UD versus MEGAHIT (Wilcox, p =0.5. Error bars are shown in cases where n≥3.

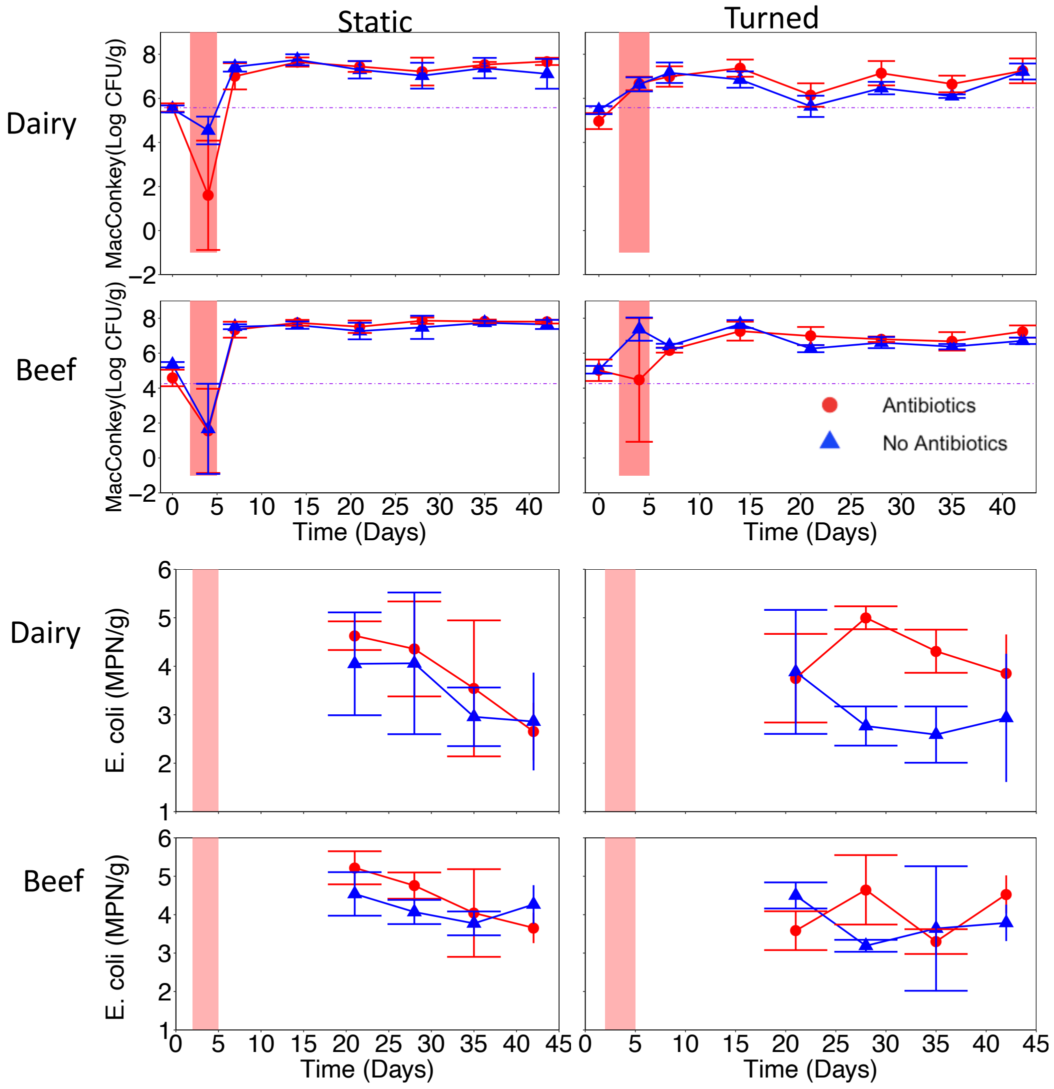

Fig. S3 Small-scale: Culture-based counts of enteric indicator targets with time in the small-scale compost. Shaded area indicates the duration of the thermophilic phase (>55ºC). The increase MacConkey counts over time was significant (p<0.004; Kruskal-Wallis). IDEXX were not consistent across conditions and varied significantly between conditions over time (p<0.003, Kruskal-Wallis). Days 21, 35 and 42 are not significantly different across Beef, Dairy, static, or turned conditions (21: p<0.189, 35: p<0.189, 42 p<0.42, Kruskal-Wallis). Day 28 was not significantly different when Dairy and Beef conditions were considered separately. (p<0.08, p<0.06, Kruskal- Wallis). Error bars represent standard deviation (n=3).

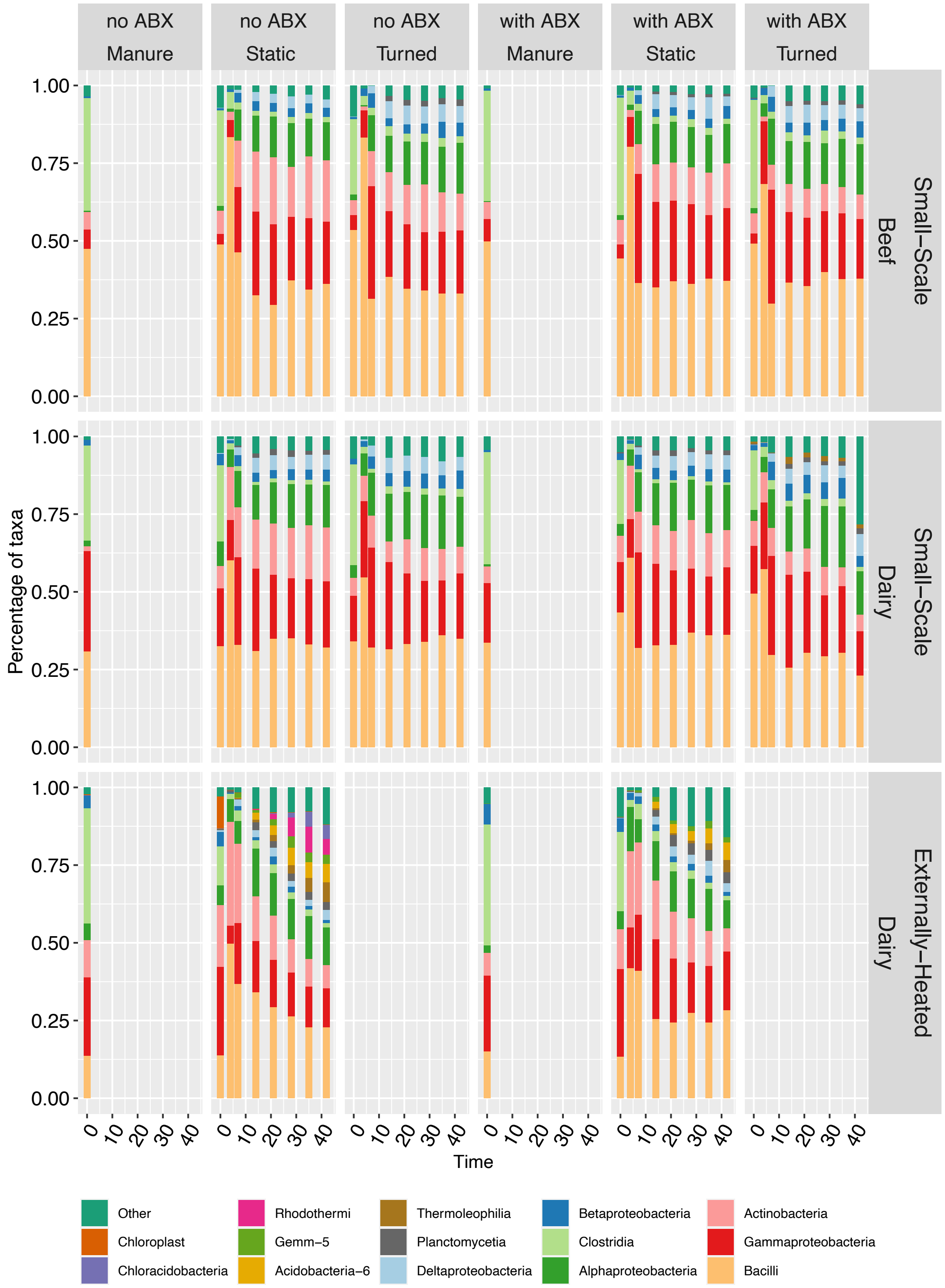

Fig. S4: 16S rRNA Amplicon sequencing of taxa at the class level

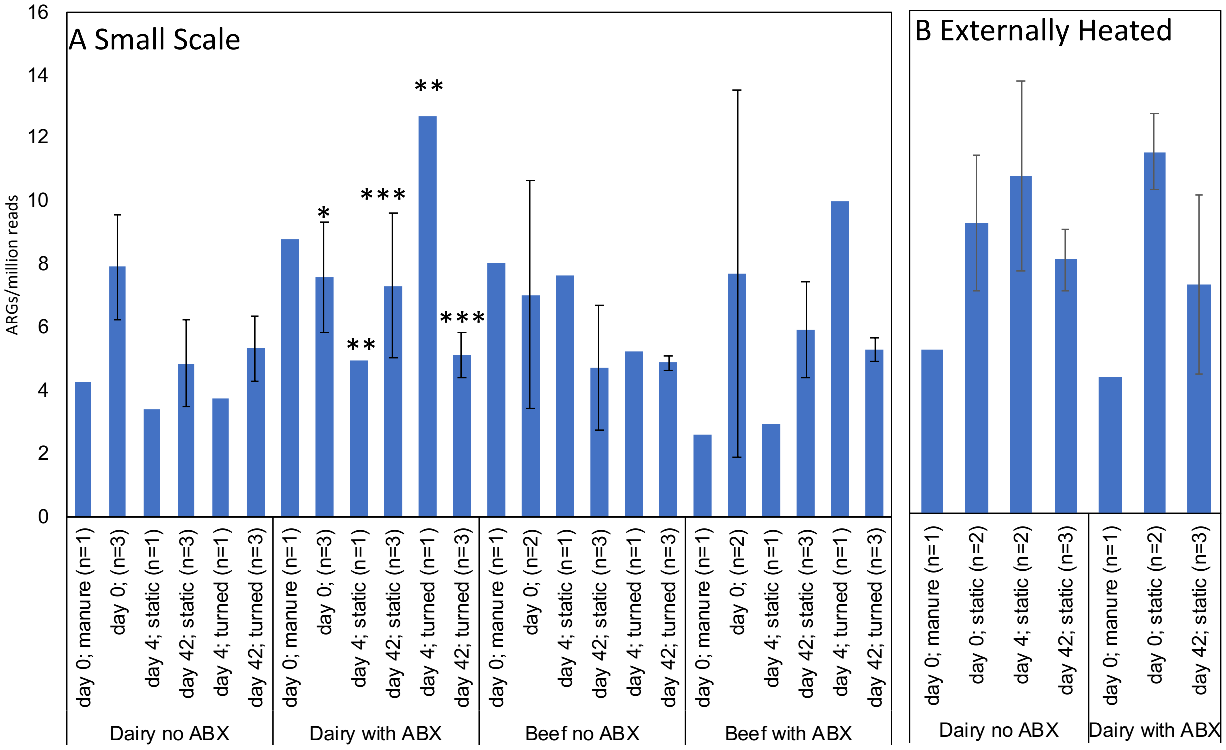

Fig. S5: Diversity as number of unique ARGs/ million reads (i.e., richness). Statistical significance was noted across the following samples with respect to time: all small-scale samples ($p = 0.04$, Kruskal Wallis), Dairy samples ($p = 0.004$, Kruskal Wallis), Dairy with ABX samples ($p = 0.05$, Kruskal Wallis). Values plotted are averages of n varying from 1-3.

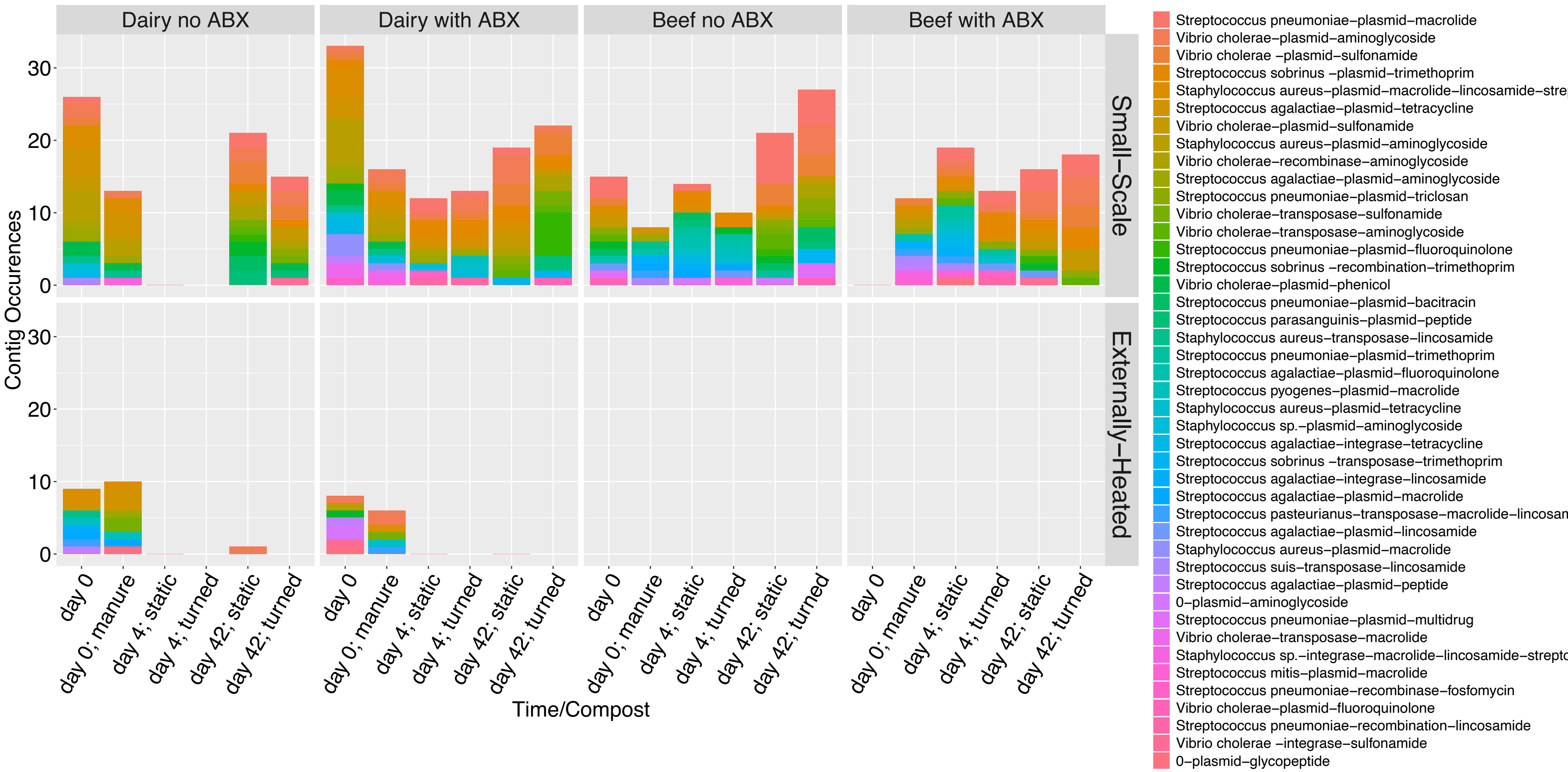

Fig. S6: ARG/MGE/Pathogen contigs detected during various stages of composting. Contigs were assembled via IDBA-UD and annotated via MetaCompare (Analysis includes contigs that occurred at least 3 times).

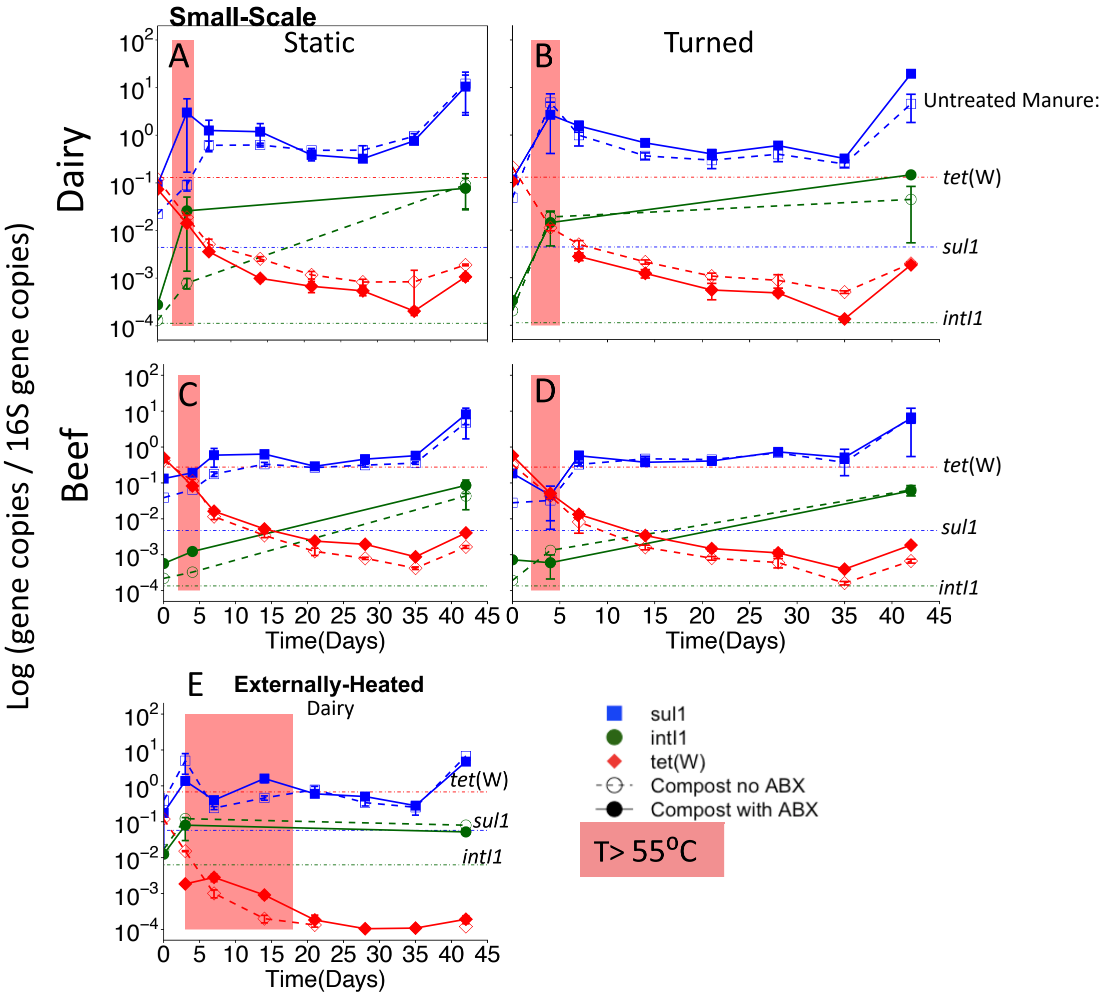

Fig. S7 A-D: Small-scale.

***sul1* normalized to 16S rRNA genes-** With respect to time: significant increase with static composted dairy with antibiotics (Kruskal-Wallis; p < 0.01), with respect to composting method: significant increase in relative abundance on day 42 with respect to raw manure on day 0 (Kruskal-Wallis; p<0.006). There was no significant difference between cow or composting methods (Static vs Turned) on day 42 (Kruskal-Wallis; p = 0.50). Error bars represent standard deviation.

***int*I1 normalized to 16S rRNA genes-** With respect to time: increased significantly with time for all compost types and methods (Kruskal-Wallis; p< 0.0001), with respect to composting method: initial values across all compost types and methods were not significantly different (p< 0.26, Kruskal-Wallis) with respect to prior antibiotic dosing: significant difference between antibiotic-treated beef versus control beef compost (Wilcox; p< 0.015).

***tet*(W) normalized to 16S rRNA genes-**With respect to time: decreased significantly for all compost types (Kruskal-Wallis; p<0.0004). With respect to composting: significant decrease in relative abundance on day 42 with respect to raw manure on day 0 (Kruskal-Wallis; p<0.0002).

With respect to antibiotic dosing and cattle type: On day 0, significantly impacted with beef manures having a higher relative abundance than dairy manures (Kruskal-Wallis; $p<0.038$). On day 42, significant impact on relative *tet*(W) abundance ($p<0.018$; Kruskal-Wallis).

Fig. S7 E Externally-Heated:

***sul*1,*intI*1,and *tet*(W) normalized to 16S rRNA genes-** With respect to time: *sul1* and *intI*1 not significantly impacted by time (Kruskal- Wallis; $p<0.79$, Kruskal- Wallis; $p<0.6$). *tet*(W) significantly decreased with time (Wilcox; $p<0.0007$).

Table S1: Metagenomic Metadata and Accessions. (Bioproject accession #: PRJNA506850, Assembled reads: MetaStorm project "Antibiotic resistance on manure and compost methods").

Trial	Cattle Type	Time	Compost	ABX?	Metagenomic Accessions	MetaStorm ID	Total Reads	Percent Assembled	Scaffold number	Average Length	N50
Externally - Heated	Dairy	0	Manure	Yes	SRR10980629	RW52	5433512.00	36.46%	246,573	710	782
	Dairy	0	Manure	No	SRR10980630	RW51	4502635.00	33.32%	240,504	675	724
	Dairy	0	Static	Yes	SRR10980632	RW49	4198838.00	27.54%	249,800	593	591
	Dairy	0	Static	Yes	SRR10980631	RW50	3113997.00	25.05%	206,818	550	550
	Dairy	42	Static	Yes	SRR10980622	RW58	8515302.00	68.09%	147,086	1186	2,135
	Dairy	42	Static	Yes	SRR10980621	RW59	9068180.00	67.62%	132,674	1229	2,276
	Dairy	42	Static	Yes	SRR10980620	RW60	9015609.00	68.55%	144,827	1195	2,083
	Dairy	0	Static	No	SRR10980634	RW47	2711024.00	23.97%	196,379	511	519
	Dairy	0	Static	No	SRR10980633	RW48	3027624.00	24.76%	214,493	516	520
	Dairy	4	Static	No	SRR10980628	RW53	7880065.00	52.36%	217,663	823	990
	Dairy	4	Static	No	SRR10980627	RW54	5107000.00	49.05%	128,316	895	1,188
	Dairy	42	Static	No	SRR10980626	RW55	8157702.00	62.12%	145,305	1150	1,950
	Dairy	42	Static	No	SRR10980625	RW56	7537130.00	60.50%	132,329	1166	2,465
	Dairy	42	Static	No	SRR10980623	RW57	7666492.00	60.56%	166,039	1092	1,757
Small-Scale	Beef	0	Manure	Yes	SRR10980329	RW12	5969539.00	56.05%	139,353	906	1,272
	Beef	0	Manure	No	SRR10980330	RW11	4302242.00	44.12%	165,731	781	910
	Beef	0	Static	Yes	SRR10980295	RW4	3157675.00	29.65%	152,041	619	616
	Beef	4	Static	Yes	SRR10980321	RW18	12518693.00	73.67%	154,382	1236	2,872
	Beef	42	Static	Yes	SRR10980311	RW26	6368772.00	50.72%	208,134	867	1,078
	Beef	42	Static	Yes	SRR10980306	RW30	6930232.00	49.53%	226,527	889	1,087
	Beef	42	Static	Yes	SRR10980301	RW34	7363326.00	52.13%	242,539	835	960
	Beef	0	Static	No	SRR10980307	RW3	3856422.00	30.44%	192,504	613	615
	Beef	4	Static	No	SRR10980322	RW17	11847415.00	72.83%	153,835	1271	3,073

Beef	42	Static	No	SRR10980312	RW25	9495097.00	52.42%	256,059	873	1,115
Beef	42	Static	No	SRR10980308	RW29	7266176.00	53.26%	183,634	915	1,193
Beef	42	Static	No	SRR10980302	RW33	7639743.00	52.41%	223,746	894	1,107
Beef	0	Turned	Yes	SRR10980637	RW8	2926848.00	28.90%	144,144	611	609
Beef	4	Turned	Yes	SRR10980316	RW22	13153502.00	77.42%	161,108	1120	2,049
Beef	42	Turned	Yes	SRR10980297	RW38	6087176.00	48.56%	210,029	896	1,117
Beef	42	Turned	Yes	SRR10980645	RW42	5181501.00	46.57%	195,647	867	1,035
Beef	42	Turned	Yes	SRR10980641	RW46	4561812.00	44.49%	189,510	818	959
Beef	0	Turned	No	SRR10980638	RW7	2562160.00	24.87%	159,282	552	541
Beef	4	Turned	No	SRR10980317	RW21	12453748.00	71.90%	177,329	1110	2,217
Beef	42	Turned	No	SRR10980298	RW37	7132271.00	55.81%	187,138	1040	1,615
Beef	42	Turned	No	SRR10980647	RW41	5762560.00	49.34%	204,578	902	1,144
Beef	42	Turned	No	SRR10980642	RW45	6305452.00	50.68%	181,041	1014	1,571
Dairy	0	Manure	Yes	SRR10980331	RW10	3838927.00	36.69%	186,308	714	789
Dairy	0	Manure	No	SRR10980636	RW9	4258214.00	37.57%	209,284	699	761
Dairy	0	Static	Yes	SRR10980327	RW14	5330678.00	29.54%	288,562	590	594
Dairy	0	Static	Yes	SRR10980319	RW2	3230141.00	25.98%	184,607	576	567
Dairy	4	Static	Yes	SRR10980323	RW16	10536778.00	71.24%	136,344	1087	1,944
Dairy	42	Static	Yes	SRR10980313	RW24	9188391.00	53.50%	262,149	1014	1,445
Dairy	42	Static	Yes	SRR10980309	RW28	6110758.00	48.04%	194,489	938	1,244
Dairy	42	Static	Yes	SRR10980304	RW32	6310100.00	45.90%	220,254	841	977
Dairy	0	Static	No	SRR10980332	RW1	3303349.00	23.54%	200,915	571	558
Dairy	0	Static	No	SRR10980328	RW13	5381237.00	27.22%	310,074	576	577
Dairy	4	Static	No	SRR10980324	RW15	13168603.00	74.60%	117,286	1207	2,743
Dairy	42	Static	No	SRR10980315	RW23	7656866.00	53.56%	230,766	976	1,334
Dairy	42	Static	No	SRR10980310	RW27	9427370.00	55.65%	235,343	1052	1,632
Dairy	42	Static	No	SRR10980305	RW31	6581279.00	45.96%	205,938	920	1,219

Dairy	0	Turned	Yes	SRR10980639	RW6	2592539.00	24.01%	163,366	529	523
Dairy	4	Turned	Yes	SRR10980318	RW20	11951698.00	75.26%	135,820	1187	2,650
Dairy	42	Turned	Yes	SRR10980299	RW36	6964777.00	47.68%	233,934	889	1,088
Dairy	42	Turned	Yes	SRR10980648	RW40	5337110.00	45.42%	195,463	850	974
Dairy	42	Turned	Yes	SRR10980643	RW44					
Dairy	0	Turned	No	SRR10980640	RW5	2568825.00	24.07%	163,901	534	524
Dairy	4	Turned	No	SRR10980320	RW19	10704079.00	72.80%	127,375	1193	2,505
Dairy	42	Turned	No	SRR10980300	RW35	5971968.00	45.49%	220,836	877	1,075
Dairy	42	Turned	No	SRR10980296	RW39	4712302.00	46.36%	171,054	886	1,082
Dairy	42	Turned	No	SRR10980644	RW43	3725575.00	38.24%	175,759	816	940

Table S2: 16SrRNA Amplicon SRAs.

16S rRNA Amplicon SRA	compost	condition	experiment	time
SRR10980547	Manure	Beef no ABX	Small Scale	0
SRR10980361	Manure	Beef no ABX	Small Scale	0
SRR10980437	Static	Beef no ABX	Small Scale	0
SRR10980592	Static	Beef no ABX	Small Scale	0
SRR10980588	Static	Beef no ABX	Small Scale	0
SRR10980355	Static	Beef no ABX	Small Scale	0
SRR10980529	Static	Beef no ABX	Small Scale	14
SRR10980524	Static	Beef no ABX	Small Scale	14
SRR10980520	Static	Beef no ABX	Small Scale	14
SRR10980349	Static	Beef no ABX	Small Scale	14
SRR10980502	Static	Beef no ABX	Small Scale	21
SRR10980498	Static	Beef no ABX	Small Scale	21
SRR10980494	Static	Beef no ABX	Small Scale	21
SRR10980346	Static	Beef no ABX	Small Scale	21
SRR10980476	Static	Beef no ABX	Small Scale	28
SRR10980472	Static	Beef no ABX	Small Scale	28
SRR10980467	Static	Beef no ABX	Small Scale	28
SRR10980450	Static	Beef no ABX	Small Scale	35
SRR10980445	Static	Beef no ABX	Small Scale	35
SRR10980441	Static	Beef no ABX	Small Scale	35
SRR10980481	Static	Beef no ABX	Small Scale	4
SRR10980575	Static	Beef no ABX	Small Scale	4
SRR10980570	Static	Beef no ABX	Small Scale	4
SRR10980353	Static	Beef no ABX	Small Scale	4
SRR10980392	Static	Beef no ABX	Small Scale	42
SRR10980348	Static	Beef no ABX	Small Scale	42
SRR10980303	Static	Beef no ABX	Small Scale	42
SRR10980557	Static	Beef no ABX	Small Scale	7
SRR10980553	Static	Beef no ABX	Small Scale	7
SRR10980546	Static	Beef no ABX	Small Scale	7
SRR10980351	Static	Beef no ABX	Small Scale	7
SRR10980593	Turned	Beef no ABX	Small Scale	0
SRR10980584	Turned	Beef no ABX	Small Scale	0

SRR10980579	Turned	Beef no ABX	Small Scale	0
SRR10980516	Turned	Beef no ABX	Small Scale	14
SRR10980511	Turned	Beef no ABX	Small Scale	14
SRR10980507	Turned	Beef no ABX	Small Scale	14
SRR10980489	Turned	Beef no ABX	Small Scale	21
SRR10980485	Turned	Beef no ABX	Small Scale	21
SRR10980480	Turned	Beef no ABX	Small Scale	21
SRR10980344	Turned	Beef no ABX	Small Scale	21
SRR10980463	Turned	Beef no ABX	Small Scale	28
SRR10980458	Turned	Beef no ABX	Small Scale	28
SRR10980454	Turned	Beef no ABX	Small Scale	28
SRR10980342	Turned	Beef no ABX	Small Scale	28
SRR10980435	Turned	Beef no ABX	Small Scale	35
SRR10980431	Turned	Beef no ABX	Small Scale	35
SRR10980427	Turned	Beef no ABX	Small Scale	35
SRR10980340	Turned	Beef no ABX	Small Scale	35
SRR10980436	Turned	Beef no ABX	Small Scale	4
SRR10980566	Turned	Beef no ABX	Small Scale	4
SRR10980562	Turned	Beef no ABX	Small Scale	4
SRR10980360	Turned	Beef no ABX	Small Scale	4
SRR10980619	Turned	Beef no ABX	Small Scale	42
SRR10980614	Turned	Beef no ABX	Small Scale	42
SRR10980610	Turned	Beef no ABX	Small Scale	42
SRR10980357	Turned	Beef no ABX	Small Scale	42
SRR10980542	Turned	Beef no ABX	Small Scale	7
SRR10980538	Turned	Beef no ABX	Small Scale	7
SRR10980533	Turned	Beef no ABX	Small Scale	7
SRR10980536	Manure	Beef with ABX	Small Scale	0
SRR10980326	Static	Beef with ABX	Small Scale	0
SRR10980591	Static	Beef with ABX	Small Scale	0
SRR10980587	Static	Beef with ABX	Small Scale	0
SRR10980528	Static	Beef with ABX	Small Scale	14
SRR10980523	Static	Beef with ABX	Small Scale	14
SRR10980519	Static	Beef with ABX	Small Scale	14
SRR10980501	Static	Beef with ABX	Small Scale	21
SRR10980497	Static	Beef with ABX	Small Scale	21
SRR10980493	Static	Beef with ABX	Small Scale	21

SRR10980475	Static	Beef with ABX	Small Scale	28
SRR10980471	Static	Beef with ABX	Small Scale	28
SRR10980466	Static	Beef with ABX	Small Scale	28
SRR10980449	Static	Beef with ABX	Small Scale	35
SRR10980444	Static	Beef with ABX	Small Scale	35
SRR10980440	Static	Beef with ABX	Small Scale	35
SRR10980470	Static	Beef with ABX	Small Scale	4
SRR10980574	Static	Beef with ABX	Small Scale	4
SRR10980569	Static	Beef with ABX	Small Scale	4
SRR10980381	Static	Beef with ABX	Small Scale	42
SRR10980337	Static	Beef with ABX	Small Scale	42
SRR10980646	Static	Beef with ABX	Small Scale	42
SRR10980556	Static	Beef with ABX	Small Scale	7
SRR10980552	Static	Beef with ABX	Small Scale	7
SRR10980545	Static	Beef with ABX	Small Scale	7
SRR10980582	Turned	Beef with ABX	Small Scale	0
SRR10980583	Turned	Beef with ABX	Small Scale	0
SRR10980578	Turned	Beef with ABX	Small Scale	0
SRR10980515	Turned	Beef with ABX	Small Scale	14
SRR10980510	Turned	Beef with ABX	Small Scale	14
SRR10980506	Turned	Beef with ABX	Small Scale	14
SRR10980488	Turned	Beef with ABX	Small Scale	21
SRR10980484	Turned	Beef with ABX	Small Scale	21
SRR10980479	Turned	Beef with ABX	Small Scale	21
SRR10980462	Turned	Beef with ABX	Small Scale	28
SRR10980457	Turned	Beef with ABX	Small Scale	28
SRR10980453	Turned	Beef with ABX	Small Scale	28
SRR10980434	Turned	Beef with ABX	Small Scale	35
SRR10980430	Turned	Beef with ABX	Small Scale	35
SRR10980426	Turned	Beef with ABX	Small Scale	35
SRR10980425	Turned	Beef with ABX	Small Scale	4
SRR10980565	Turned	Beef with ABX	Small Scale	4
SRR10980561	Turned	Beef with ABX	Small Scale	4
SRR10980618	Turned	Beef with ABX	Small Scale	42
SRR10980613	Turned	Beef with ABX	Small Scale	42
SRR10980609	Turned	Beef with ABX	Small Scale	42
SRR10980541	Turned	Beef with ABX	Small Scale	7
SRR10980537	Turned	Beef with ABX	Small Scale	7
SRR10980532	Turned	Beef with ABX	Small Scale	7
SRR10980571	Manure	Dairy no ABX	Small Scale	0

SRR10980549	Static	Dairy no ABX	Small Scale	0
SRR10980525	Static	Dairy no ABX	Small Scale	0
SRR10980590	Static	Dairy no ABX	Small Scale	0
SRR10980362	Static	Dairy no ABX	Small Scale	0
SRR10980531	Static	Dairy no ABX	Small Scale	14
SRR10980527	Static	Dairy no ABX	Small Scale	14
SRR10980522	Static	Dairy no ABX	Small Scale	14
SRR10980505	Static	Dairy no ABX	Small Scale	21
SRR10980500	Static	Dairy no ABX	Small Scale	21
SRR10980496	Static	Dairy no ABX	Small Scale	21
SRR10980478	Static	Dairy no ABX	Small Scale	28
SRR10980474	Static	Dairy no ABX	Small Scale	28
SRR10980469	Static	Dairy no ABX	Small Scale	28
SRR10980343	Static	Dairy no ABX	Small Scale	28
SRR10980452	Static	Dairy no ABX	Small Scale	35
SRR10980447	Static	Dairy no ABX	Small Scale	35
SRR10980443	Static	Dairy no ABX	Small Scale	35
SRR10980341	Static	Dairy no ABX	Small Scale	35
SRR10980503	Static	Dairy no ABX	Small Scale	4
SRR10980577	Static	Dairy no ABX	Small Scale	4
SRR10980573	Static	Dairy no ABX	Small Scale	4
SRR10980352	Static	Dairy no ABX	Small Scale	4
SRR10980414	Static	Dairy no ABX	Small Scale	42
SRR10980370	Static	Dairy no ABX	Small Scale	42
SRR10980325	Static	Dairy no ABX	Small Scale	42
SRR10980358	Static	Dairy no ABX	Small Scale	42
SRR10980559	Static	Dairy no ABX	Small Scale	7
SRR10980555	Static	Dairy no ABX	Small Scale	7
SRR10980551	Static	Dairy no ABX	Small Scale	7
SRR10980615	Turned	Dairy no ABX	Small Scale	0
SRR10980586	Turned	Dairy no ABX	Small Scale	0
SRR10980581	Turned	Dairy no ABX	Small Scale	0
SRR10980354	Turned	Dairy no ABX	Small Scale	0
SRR10980518	Turned	Dairy no ABX	Small Scale	14
SRR10980513	Turned	Dairy no ABX	Small Scale	14
SRR10980509	Turned	Dairy no ABX	Small Scale	14
SRR10980347	Turned	Dairy no ABX	Small Scale	14
SRR10980491	Turned	Dairy no ABX	Small Scale	21
SRR10980487	Turned	Dairy no ABX	Small Scale	21
SRR10980483	Turned	Dairy no ABX	Small Scale	21

SRR10980345	Turned	Dairy no ABX	Small Scale	21
SRR10980465	Turned	Dairy no ABX	Small Scale	28
SRR10980461	Turned	Dairy no ABX	Small Scale	28
SRR10980456	Turned	Dairy no ABX	Small Scale	28
SRR10980439	Turned	Dairy no ABX	Small Scale	35
SRR10980433	Turned	Dairy no ABX	Small Scale	35
SRR10980429	Turned	Dairy no ABX	Small Scale	35
SRR10980459	Turned	Dairy no ABX	Small Scale	4
SRR10980568	Turned	Dairy no ABX	Small Scale	4
SRR10980564	Turned	Dairy no ABX	Small Scale	4
SRR10980635	Turned	Dairy no ABX	Small Scale	42
SRR10980617	Turned	Dairy no ABX	Small Scale	42
SRR10980612	Turned	Dairy no ABX	Small Scale	42
SRR10980544	Turned	Dairy no ABX	Small Scale	7
SRR10980540	Turned	Dairy no ABX	Small Scale	7
SRR10980535	Turned	Dairy no ABX	Small Scale	7
SRR10980350	Turned	Dairy no ABX	Small Scale	7
SRR10980560	Manure	Dairy with ABX	Small Scale	0
SRR10980548	Static	Dairy with ABX	Small Scale	0
SRR10980514	Static	Dairy with ABX	Small Scale	0
SRR10980589	Static	Dairy with ABX	Small Scale	0
SRR10980530	Static	Dairy with ABX	Small Scale	14
SRR10980526	Static	Dairy with ABX	Small Scale	14
SRR10980521	Static	Dairy with ABX	Small Scale	14
SRR10980504	Static	Dairy with ABX	Small Scale	21
SRR10980499	Static	Dairy with ABX	Small Scale	21
SRR10980495	Static	Dairy with ABX	Small Scale	21
SRR10980477	Static	Dairy with ABX	Small Scale	28
SRR10980473	Static	Dairy with ABX	Small Scale	28
SRR10980468	Static	Dairy with ABX	Small Scale	28
SRR10980451	Static	Dairy with ABX	Small Scale	35
SRR10980446	Static	Dairy with ABX	Small Scale	35
SRR10980442	Static	Dairy with ABX	Small Scale	35
SRR10980492	Static	Dairy with ABX	Small Scale	4
SRR10980576	Static	Dairy with ABX	Small Scale	4
SRR10980572	Static	Dairy with ABX	Small Scale	4
SRR10980403	Static	Dairy with ABX	Small Scale	42
SRR10980359	Static	Dairy with ABX	Small Scale	42
SRR10980314	Static	Dairy with ABX	Small Scale	42
SRR10980558	Static	Dairy with ABX	Small Scale	7

SRR10980554	Static	Dairy with ABX	Small Scale	7
SRR10980550	Static	Dairy with ABX	Small Scale	7
SRR10980604	Turned	Dairy with ABX	Small Scale	0
SRR10980585	Turned	Dairy with ABX	Small Scale	0
SRR10980580	Turned	Dairy with ABX	Small Scale	0
SRR10980517	Turned	Dairy with ABX	Small Scale	14
SRR10980512	Turned	Dairy with ABX	Small Scale	14
SRR10980508	Turned	Dairy with ABX	Small Scale	14
SRR10980490	Turned	Dairy with ABX	Small Scale	21
SRR10980486	Turned	Dairy with ABX	Small Scale	21
SRR10980482	Turned	Dairy with ABX	Small Scale	21
SRR10980464	Turned	Dairy with ABX	Small Scale	28
SRR10980460	Turned	Dairy with ABX	Small Scale	28
SRR10980455	Turned	Dairy with ABX	Small Scale	28
SRR10980438	Turned	Dairy with ABX	Small Scale	35
SRR10980432	Turned	Dairy with ABX	Small Scale	35
SRR10980428	Turned	Dairy with ABX	Small Scale	35
SRR10980448	Turned	Dairy with ABX	Small Scale	4
SRR10980567	Turned	Dairy with ABX	Small Scale	4
SRR10980563	Turned	Dairy with ABX	Small Scale	4
SRR10980624	Turned	Dairy with ABX	Small Scale	42
SRR10980616	Turned	Dairy with ABX	Small Scale	42
SRR10980611	Turned	Dairy with ABX	Small Scale	42
SRR10980543	Turned	Dairy with ABX	Small Scale	7
SRR10980539	Turned	Dairy with ABX	Small Scale	7
SRR10980534	Turned	Dairy with ABX	Small Scale	7
SRR10980603	Manure	Dairy no ABX	Externally Heated	0
SRR10980356	Manure	Dairy no ABX	Externally Heated	0
SRR10980608	Static	Dairy no ABX	Externally Heated	0
SRR10980607	Static	Dairy no ABX	Externally Heated	0
SRR10980424	Static	Dairy no ABX	Externally Heated	0
SRR10980411	Static	Dairy no ABX	Externally Heated	14
SRR10980410	Static	Dairy no ABX	Externally Heated	14
SRR10980409	Static	Dairy no ABX	Externally Heated	14
SRR10980405	Static	Dairy no ABX	Externally Heated	21
SRR10980404	Static	Dairy no ABX	Externally Heated	21
SRR10980402	Static	Dairy no ABX	Externally Heated	21
SRR10980398	Static	Dairy no ABX	Externally Heated	28
SRR10980397	Static	Dairy no ABX	Externally Heated	28
SRR10980396	Static	Dairy no ABX	Externally Heated	28

SRR10980336	Static	Dairy no ABX	Externally Heated	28
SRR10980422	Static	Dairy no ABX	Externally Heated	3
SRR10980391	Static	Dairy no ABX	Externally Heated	35
SRR10980390	Static	Dairy no ABX	Externally Heated	35
SRR10980389	Static	Dairy no ABX	Externally Heated	35
SRR10980601	Static	Dairy no ABX	Externally Heated	4
SRR10980600	Static	Dairy no ABX	Externally Heated	4
SRR10980599	Static	Dairy no ABX	Externally Heated	42
SRR10980598	Static	Dairy no ABX	Externally Heated	42
SRR10980597	Static	Dairy no ABX	Externally Heated	42
SRR10980418	Static	Dairy no ABX	Externally Heated	7
SRR10980417	Static	Dairy no ABX	Externally Heated	7
SRR10980416	Static	Dairy no ABX	Externally Heated	7
SRR10980602	Manure	Dairy with ABX	Externally Heated	0
SRR10980606	Static	Dairy with ABX	Externally Heated	0
SRR10980605	Static	Dairy with ABX	Externally Heated	0
SRR10980423	Static	Dairy with ABX	Externally Heated	0
SRR10980408	Static	Dairy with ABX	Externally Heated	14
SRR10980407	Static	Dairy with ABX	Externally Heated	14
SRR10980406	Static	Dairy with ABX	Externally Heated	14
SRR10980338	Static	Dairy with ABX	Externally Heated	14
SRR10980401	Static	Dairy with ABX	Externally Heated	21
SRR10980400	Static	Dairy with ABX	Externally Heated	21
SRR10980399	Static	Dairy with ABX	Externally Heated	21
SRR10980395	Static	Dairy with ABX	Externally Heated	28
SRR10980394	Static	Dairy with ABX	Externally Heated	28
SRR10980393	Static	Dairy with ABX	Externally Heated	28
SRR10980421	Static	Dairy with ABX	Externally Heated	3
SRR10980420	Static	Dairy with ABX	Externally Heated	3
SRR10980419	Static	Dairy with ABX	Externally Heated	3
SRR10980339	Static	Dairy with ABX	Externally Heated	3
SRR10980388	Static	Dairy with ABX	Externally Heated	35
SRR10980387	Static	Dairy with ABX	Externally Heated	35
SRR10980386	Static	Dairy with ABX	Externally Heated	35
SRR10980335	Static	Dairy with ABX	Externally Heated	35
SRR10980596	Static	Dairy with ABX	Externally Heated	42
SRR10980595	Static	Dairy with ABX	Externally Heated	42
SRR10980594	Static	Dairy with ABX	Externally Heated	42
SRR10980415	Static	Dairy with ABX	Externally Heated	7
SRR10980413	Static	Dairy with ABX	Externally Heated	7

SRR10980412	Static	Dairy with ABX	Externally Heated	7

Table S3: Clinically-Relevant ARGs.

CARB-1	CARB-3	CTX-M-104	CTX-M-116	CTX-M-132	CTX-M-152	CTX-M-22
CARB-10	CARB-4	CTX-M-105	CTX-M-117	CTX-M-134	CTX-M-155	CTX-M-23
CARB-12	CARB-5	CTX-M-106	CTX-M-12	CTX-M-136	CTX-M-156	CTX-M-24
CARB-14	CARB-6	CTX-M-107	CTX-M-121	CTX-M-137	CTX-M-157	CTX-M-25
CARB-16	CARB-7	CTX-M-108	CTX-M-122	CTX-M-139	CTX-M-158	CTX-M-26
CARB-17	CARB-8	CTX-M-109	CTX-M-123	CTX-M-14	CTX-M-159	CTX-M-27
CARB-18	CARB-9	CTX-M-11	CTX-M-124	CTX-M-141	CTX-M-16	CTX-M-28
CARB-19	CTX-M-1	CTX-M-110	CTX-M-125	CTX-M-142	CTX-M-160	CTX-M-29
CARB-2	CTX-M-10	CTX-M-111	CTX-M-126	CTX-M-144	CTX-M-17	CTX-M-3
CARB-20	CTX-M-100	CTX-M-112	CTX-M-129	CTX-M-147	CTX-M-19	CTX-M-30
CARB-21	CTX-M-101	CTX-M-113	CTX-M-13	CTX-M-148	CTX-M-2	CTX-M-31
CARB-22	CTX-M-102	CTX-M-114	CTX-M-130	CTX-M-15	CTX-M-20	CTX-M-32
CARB-23	CTX-M-103	CTX-M-115	CTX-M-131	CTX-M-151	CTX-M-21	CTX-M-33
CTX-M-34	CTX-M-76	GES-3	OXA-100	OXA-15	OXA-203	OXA-249
CTX-M-35	CTX-M-77	GES-4	OXA-101	OXA-150	OXA-204	OXA-25
CTX-M-36	CTX-M-78	GES-5	OXA-104	OXA-16	OXA-205	OXA-250
CTX-M-37	CTX-M-79	GES-6	OXA-106	OXA-160	OXA-206	OXA-251
CTX-M-38	CTX-M-8	GES-7	OXA-107	OXA-161	OXA-207	OXA-253
CTX-M-39	CTX-M-80	GES-8	OXA-108	OXA-162	OXA-208	OXA-254
CTX-M-4	CTX-M-81	GES-9	OXA-109	OXA-163	OXA-209	OXA-255
CTX-M-40	CTX-M-82	KPC-10	OXA-11	OXA-164	OXA-21	OXA-256
CTX-M-41	CTX-M-83	KPC-11	OXA-110	OXA-165	OXA-210	OXA-257
CTX-M-42	CTX-M-84	KPC-12	OXA-111	OXA-166	OXA-211	OXA-258
CTX-M-43	CTX-M-85	KPC-13	OXA-112	OXA-167	OXA-212	OXA-26
CTX-M-44	CTX-M-86	KPC-14	OXA-113	OXA-168	OXA-213	OXA-27
CTX-M-45	CTX-M-87	KPC-15	OXA-114a	OXA-169	OXA-214	OXA-278
CTX-M-46	CTX-M-88	KPC-16	OXA-115	OXA-17	OXA-215	OXA-28
CTX-M-47	CTX-M-89	KPC-17	OXA-116	OXA-170	OXA-216	OXA-29
CTX-M-48	CTX-M-9	KPC-19	OXA-117	OXA-171	OXA-217	OXA-3
CTX-M-49	CTX-M-90	KPC-2	OXA-118	OXA-172	OXA-219	OXA-309
CTX-M-5	CTX-M-91	KPC-22	OXA-119	OXA-173	OXA-22	OXA-31
CTX-M-50	CTX-M-92	KPC-3	OXA-12	OXA-174	OXA-223	OXA-312
CTX-M-51	CTX-M-93	KPC-4	OXA-120	OXA-175	OXA-224	OXA-313
CTX-M-52	CTX-M-94	KPC-5	OXA-121	OXA-176	OXA-225	OXA-314
CTX-M-53	CTX-M-95	KPC-6	OXA-128	OXA-177	OXA-226	OXA-315
CTX-M-54	CTX-M-96	KPC-7	OXA-129	OXA-178	OXA-228	OXA-316
CTX-M-55	CTX-M-98	KPC-8	OXA-13	OXA-179	OXA-229	OXA-317
CTX-M-56	CTX-M-99	KPC-9	OXA-130	OXA-18	OXA-23	OXA-32
CTX-M-58	GES-1	MCR-1	OXA-131	OXA-180	OXA-230	OXA-320

CTX-M-59	GES-10	mecA	OXA-132	OXA-181	OXA-231	OXA-322
CTX-M-6	GES-11	NDM-1	OXA-133	OXA-182	OXA-232	OXA-323
CTX-M-60	GES-12	NDM-10	OXA-134	OXA-183	OXA-233	OXA-324
CTX-M-61	GES-13	NDM-12	OXA-136	OXA-184	OXA-235	OXA-325
CTX-M-62	GES-14	NDM-13	OXA-137	OXA-19	OXA-236	OXA-326
CTX-M-63	GES-15	NDM-14	OXA-138	OXA-192	OXA-237	OXA-327
CTX-M-64	GES-16	NDM-17	OXA-139	OXA-194	OXA-239	OXA-328
CTX-M-65	GES-17	NDM-2	OXA-14	OXA-195	OXA-24	OXA-329
CTX-M-66	GES-18	NDM-3	OXA-141	OXA-196	OXA-240	OXA-33
CTX-M-67	GES-19	NDM-4	OXA-142	OXA-197	OXA-241	OXA-330
CTX-M-68	GES-2	NDM-5	OXA-143	OXA-198	OXA-242	OXA-331
CTX-M-69	GES-20	NDM-6	OXA-144	OXA-199	OXA-243	OXA-332
CTX-M-7	GES-21	NDM-7	OXA-145	OXA-2	OXA-244	OXA-333
CTX-M-71	GES-22	NDM-8	OXA-146	OXA-20	OXA-245	OXA-334
CTX-M-72	GES-23	NDM-9	OXA-147	OXA-200	OXA-246	OXA-335
CTX-M-74	GES-24	OXA-1	OXA-148	OXA-201	OXA-247	OXA-338
CTX-M-75	GES-26	OXA-10	OXA-149	OXA-202	OXA-248	OXA-34
OXA-347	OXA-397	OXA-71	QnrS8	SHV-149	SHV-26	SHV-69
OXA-348	OXA-398	OXA-72	QnrS9	SHV-15	SHV-27	SHV-7
OXA-349	OXA-4	OXA-73	SHV-1	SHV-150	SHV-28	SHV-70
OXA-35	OXA-415	OXA-74	SHV-100	SHV-151	SHV-29	SHV-71
OXA-350	OXA-418	OXA-75	SHV-101	SHV-152	SHV-2A	SHV-72
OXA-351	OXA-42	OXA-76	SHV-102	SHV-153	SHV-3	SHV-73
OXA-352	OXA-420	OXA-77	SHV-103	SHV-154	SHV-30	SHV-74
OXA-353	OXA-421	OXA-78	SHV-104	SHV-155	SHV-31	SHV-75
OXA-354	OXA-422	OXA-79	SHV-105	SHV-156	SHV-32	SHV-76
OXA-355	OXA-423	OXA-80	SHV-106	SHV-157	SHV-33	SHV-77
OXA-356	OXA-424	OXA-82	SHV-107	SHV-158	SHV-34	SHV-78
OXA-357	OXA-425	OXA-83	SHV-108	SHV-159	SHV-35	SHV-79
OXA-358	OXA-426	OXA-84	SHV-109	SHV-16	SHV-36	SHV-8
OXA-359	OXA-43	OXA-85	SHV-11	SHV-160	SHV-37	SHV-80
OXA-36	OXA-435	OXA-86	SHV-110	SHV-161	SHV-38	SHV-81
OXA-360	OXA-45	OXA-87	SHV-111	SHV-162	SHV-39	SHV-82
OXA-361	OXA-454	OXA-88	SHV-112	SHV-163	SHV-40	SHV-83
OXA-362	OXA-46	OXA-89	SHV-119	SHV-164	SHV-41	SHV-84
OXA-363	OXA-47	OXA-9	SHV-12	SHV-165	SHV-42	SHV-85
OXA-365	OXA-48	OXA-90	SHV-120	SHV-167	SHV-43	SHV-86
OXA-366	OXA-49	OXA-91	SHV-121	SHV-168	SHV-44	SHV-89
OXA-368	OXA-5	OXA-92	SHV-122	SHV-172	SHV-45	SHV-9
OXA-37	OXA-50	OXA-93	SHV-123	SHV-173	SHV-46	SHV-92
OXA-370	OXA-51	OXA-94	SHV-124	SHV-178	SHV-48	SHV-93
OXA-371	OXA-53	OXA-95	SHV-125	SHV-179	SHV-49	SHV-94
OXA-374	OXA-54	OXA-96	SHV-126	SHV-18	SHV-5	SHV-95

OXA-375	OXA-55	OXA-97	SHV-127	SHV-180	SHV-50	SHV-96
OXA-376	OXA-56	OXA-98	SHV-128	SHV-181	SHV-51	SHV-97
OXA-377	OXA-57	OXA-99	SHV-129	SHV-182	SHV-52	SHV-98
OXA-378	OXA-58	QnrA1	SHV-13	SHV-183	SHV-53	SHV-99
OXA-379	OXA-59	QnrA2	SHV-133	SHV-185	SHV-55	TEM-1
OXA-380	OXA-60	QnrA3	SHV-134	SHV-186	SHV-56	TEM-10
OXA-381	OXA-61	QnrA4	SHV-135	SHV-187	SHV-57	TEM-101
OXA-382	OXA-62	QnrA5	SHV-137	SHV-188	SHV-59	TEM-102
OXA-383	OXA-63	QnrA6	SHV-14	SHV-189	SHV-6	TEM-104
OXA-384	OXA-64	QnrA7	SHV-140	SHV-19	SHV-60	TEM-105
OXA-385	OXA-65	QnrS1	SHV-141	SHV-2	SHV-61	TEM-106
OXA-386	OXA-66	QnrS2	SHV-142	SHV-20	SHV-62	TEM-107
OXA-387	OXA-67	QnrS3	SHV-143	SHV-21	SHV-63	TEM-108
OXA-388	OXA-68	QnrS4	SHV-144	SHV-22	SHV-64	TEM-109
OXA-389	OXA-69	QnrS5	SHV-145	SHV-23	SHV-65	TEM-11
OXA-390	OXA-7	QnrS6	SHV-147	SHV-24	SHV-66	TEM-110
OXA-391	OXA-70	QnrS7	SHV-148	SHV-25	SHV-67	TEM-111
TEM-112	TEM-155	TEM-207	TEM-72	VIM-27		
TEM-113	TEM-156	TEM-208	TEM-73	VIM-28		
TEM-114	TEM-157	TEM-209	TEM-75	VIM-29		
TEM-115	TEM-158	TEM-21	TEM-76	VIM-3		
TEM-116	TEM-159	TEM-211	TEM-77	VIM-30		
TEM-117	TEM-16	TEM-213	TEM-78	VIM-31		
TEM-118	TEM-160	TEM-214	TEM-79	VIM-32		
TEM-12	TEM-162	TEM-215	TEM-8	VIM-33		
TEM-120	TEM-163	TEM-216	TEM-80	VIM-34		
TEM-121	TEM-164	TEM-217	TEM-81	VIM-35		
TEM-122	TEM-166	TEM-219	TEM-82	VIM-36		
TEM-123	TEM-167	TEM-22	TEM-83	VIM-37		
TEM-124	TEM-168	TEM-220	TEM-84	VIM-38		
TEM-125	TEM-169	TEM-24	TEM-85	VIM-39		
TEM-126	TEM-17	TEM-26	TEM-86	VIM-4		
TEM-127	TEM-171	TEM-28	TEM-87	VIM-42		
TEM-128	TEM-176	TEM-29	TEM-88	VIM-43		
TEM-129	TEM-177	TEM-3	TEM-89	VIM-5		
TEM-130	TEM-178	TEM-30	TEM-90	VIM-6		
TEM-131	TEM-182	TEM-33	TEM-91	VIM-7		
TEM-132	TEM-183	TEM-34	TEM-92	VIM-8		
TEM-133	TEM-184	TEM-4	TEM-93	VIM-9		
TEM-134	TEM-185	TEM-40	TEM-94			
TEM-135	TEM-186	TEM-42	TEM-95			
TEM-136	TEM-187	TEM-43	TEM-96			
TEM-137	TEM-188	TEM-45	vanA			

TEM-138	TEM-189	TEM-47	VIM-1
TEM-139	TEM-19	TEM-48	VIM-10
TEM-141	TEM-190	TEM-49	VIM-11
TEM-142	TEM-191	TEM-52	VIM-12
TEM-143	TEM-192	TEM-53	VIM-13
TEM-144	TEM-193	TEM-54	VIM-14
TEM-145	TEM-194	TEM-55	VIM-15
TEM-146	TEM-195	TEM-57	VIM-16
TEM-147	TEM-196	TEM-59	VIM-17
TEM-148	TEM-197	TEM-6	VIM-18
TEM-149	TEM-198	TEM-60	VIM-19
TEM-15	TEM-199	TEM-63	VIM-2
TEM-150	TEM-2	TEM-67	VIM-20
TEM-151	TEM-20	TEM-68	VIM-23
TEM-152	TEM-201	TEM-7	VIM-24
TEM-153	TEM-205	TEM-70	VIM-25
TEM-154	TEM-206	TEM-71	VIM-26

Table S4: Externally Heated Condition plated on MacConkey agar.

Treatment	Time ()	Log CFU/g
Antibiotic Compost	0	6.3 ± 0.4
	3	1.7 ± 1.4
Antibiotic Manure	0	7.3 ± 0.01
	7	7.3 ± 0.07
Control Compost	0	5.6 ± 0.2
	3	3.5 ± 0.3
Control Manure	0	6.7 ± 0.5
	7	6.8 ± 0.5

Table S5: Externally Heated: Measured Dry Matter, Nitrogen, Carbon and pirlimycin concentrations

	Manure		% Dry Matter	% Nitrogen	% Carbon	PIR (ng/g dry weight)
1	Control	0	54.9	1.4	17.2	128.8
2	Control	0	54.3	1.3	24.5	119.3
3	Control	0	51	1.8	38.0	135.0
4	Antibiotic	0	50.7	1.8	50.2	136.2

5	Antibiotic	0	48.9	1.9	49.0	139.3
6	Antibiotic	0	47.6	1.8	50.0	143.9
1	Control	3	48.2	1.3	36.8	127.1
2	Control	3	45.9	1.4	37.8	138.1
3	Control	3	46.8	1.2	40.0	127.4
4	Antibiotic	3	46.4	1.7	54.3	130.1
5	Antibiotic	3	51.6	1.7	47.3	137.5
6	Antibiotic	3	52.7	1.8	48.1	128.0
1	Control	35	59.4	1.1	23.6	0.9
2	Control	35	53.9	1.1	21.7	1.1
3	Control	35	57.1	1.1	25.8	ND
4	Antibiotic	35	63.4	1.6	34.2	0.8
5	Antibiotic	35	66.0	1.7	35.2	0.9
6	Antibiotic	35	63.6	1.7	39.2	

Table S6 : Significant comparisons.

Experiment	Gene	Comparison	Test	P value
Small-scale	16S rRNA	4 by Manure Type	Kruskal Wallis	0.01
	16S rRNA	7 by Manure Type	Kruskal Wallis	0.01
	16S rRNA	4: All conditions static	Kruskal Wallis	0.04
	16S rRNA	4: All conditions turned	Kruskal Wallis	0.03
	16S rRNA	4: static dairy vs static beef	Kruskal Wallis	0.004
	16S rRNA	4: turned dairy vs static beef	Kruskal Wallis	0.002
	tet(W)	0 by Manure Type	Kruskal Wallis	1.58 e-10
	tet(W)	Antibiotic vs non antibiotic	Kruskal Wallis	0.03
	tet(W)	0 vs 42 for each Manure Type	Wilcox	0.004 – 0.02
	sul1	0 by Manure Type	Kruskal Wallis	0.008
	sul1	0 compost vs 42 for all manure types	Wilcox	0.0004 – 0.03
	sul1	42 by manure type	Kruskal Wallis	0.001
	intI1	0 compost vs 42 for all manure types	Wilcox	0.0004 –0.008
	intI1	0 by Manure Type	Kruskal-Wallis	0.008
	intI1	42 by manure type	Kruskal-Wallis	0.004
Externally-heated	*tet(W)*	0 by Manure Type	Kruskal-Wallis	0.01
	tet(W)	42 by manure type	Kruskal-Wallis	0.02
	tet(W)	0 vs 42 Yes	Wilcox	0.004
	tet(W)	0 vs 42 No	Wilcox	0.008

	sul1	0 by Manure Type	Kruskal-Wallis	0.008
	sul1	0 vs 42 Yes	Wilcox	0.004
	intI1	0 by Manure Type	Kruskal-Wallis	0.008
	intI1	42 by manure type	Kruskal-Wallis	0.04

References:

1. Guo F, Zhang T. Biases during DNA extraction of activated sludge samples revealed by high throughput sequencing. Applied Microbiology and Biotechnology. 2013;97(10):4607-16.
